# Adverse events following immunization during COVID-19 mass vaccination campaigns in the Democratic Republic of Congo: Findings from active safety surveillance

**DOI:** 10.1371/journal.pone.0309628

**Published:** 2026-07-10

**Authors:** Kizito Kayumba, Alemayehu Duga, Mosoka Papa Fallah, Claire Tshidibi, Nicky Lubaki, Barnabas Coulibaly, Benjamin Djoudalbaye, Efire Nora Sylvana, Senga Lucy Sembuche, Tedi Tilahun, Murtala Jibril, Carlos Kilowe, Aminu Kuba, Antoine Mafwila Lusala, Nebiyu Dereje, Nicaise Ndembi, Tajudeen Raji, Ngashi Ngongo

**Affiliations:** 1 Africa Centres for Disease Control and Prevention (Africa CDC), Addis Ababa, Ethiopia; 2 Ministry of Health, Kinshasa, Democratic Republic of Congo; Universite Ziane Achour de Djelfa Faculte des Sciences de la Nature et de la Vie, ALGERIA

## Abstract

**Introduction:**

Post-marketing safety surveillance is essential for ensuring vaccine safety and maintaining public trust, particularly in African settings where evidence on the safety of COVID–19 vaccines remains limited. This study aimed to determine the overall incidence and types of AEFIs, as well as the factors associated with their occurrence, during COVI-19 mass vaccination campaigns in Democratic Republic of Congo.

**Methods:**

From December 1–29, 2023, a prospective safety surveillance study was conducted in Kinshasa Province. Participants were surveyed through phone calls from day 1–28 following COVID-19 vaccine administration. AEFI incidence rates were calculated by type of vaccine, sex, number of dose and age group. Factors associated with AEFIs were identified using multivariable logistic regression models, expressed by adjusted odds ratio and its 95% confidence interval.

**Results:**

The study included 4766 participants. Their median [IQR] age was 36 [27−48] years and 2503 (53%) were males, 94.63% received the J&J vaccine while 256 (5.37%) received the BNT162b2 vaccine. Incidence of AEFIs was 23.75% (95%CI: 22.54%−24.99%). AEFIs mostly reported were fever (9.61%, 95%CI: 8.88%−10.48%), injection site pain (9.00%, 95%CI: 8.20%−9.85%), headache (4.11%, 95%CI: 3.57%−4.72%), stiffness (1.51%, 95%CI: 1.18%−1.89%) and myalgia (1.15%, 95%CI: 0.87%−1.49%). The incidence of AEFIs was higher for the BNT162b2 vaccine at 34.48% (95% CI: 28.57%−40.54%) vs. 23.15% (95% CI: 21.92%−24.41%) for the J&J vaccine. Compared to participants aged 18−59 years, those under 18 years old were associated with decreased odds of reporting any AEFI (aOR= 0.26, 95%CI: 0.13–0.88) and injection site pain (aOR=0.18, 95%CI: 0.05–0.75). Those aged 60 years and older were associated with decreased odds of reporting any AEFI (aOR= 0.78, 95%CI: 0.62–0.99) and fever (aOR=0. 63, 95%CI: 0.43–0.92).

**Conclusions:**

Approximately one-quarter of participants reported AEFI. The observed association with vaccine type and age underscores the need for systematic vaccine safety monitoring in the population. This is critical for guiding future vaccination strategies tailored to individuals who may be more susceptible to AEFIs.

## Introduction and setting

Vaccines approved for use in national immunization programmes (NIPs) are deemed safe and effective based on robust evidence from randomized controlled clinical trials (RCTs) [1]. However, despite the rigorous evaluation conducted during clinical development, rare adverse events (AEs) may go undetected due to the limited size and duration of these trials. Therefore, post-marketing safety surveillance is essential after the introduction of vaccines like those for COVID-19 into public immunization programs to identify rare and previously undocumented adverse events (AEs). For instance, the non-replicating vector COVID-19 vaccines such as those from AstraZeneca and Johnson & Johnson have been linked to rare cases of central venous sinus and thrombocytopenia, occurring at an estimated rate of approximately 4 cases per million vaccinations [2]. Similarly, messenger RNA (mRNA) vaccines, such as the Pfizer-BioNTech BNT162b2, have been associated with rare side effects including anaphylaxis and myocarditis [3].

The need for robust post-market surveillance is particularly critical in Africa, as African populations were underrepresented in the initial COVID-19 vaccine RCTs. Consequently, the safety profile of these vaccines in this demographic can only be fully elucidated through dedicated safety monitoring after rollout. Maintaining public trust is paramount to the success of any NIP, especially since vaccines are administered predominantly to healthy individuals [4]. Thus, systematic vaccine safety surveillance is vital not only for ensuring the safety but also for sustaining public confidence. Such surveillance generates critical data on the incidence, distribution, and risk factors for both expected and unexpected adverse events following immunization (AEFIs) [5]. In line with World Health Organization (WHO) guidelines, all adverse events (AEs) that concern caregivers or incur health costs should be reported, regardless of severity [6].

This study aimed to investigate the occurrence of AEFIs during the COVID-19 mass vaccination campaigns in the Democratic Republic of Congo (DRC). By analyzing AEFI data collected from these campaigns, we sought to generate evidence on the safety profile of the vaccines in this population, thereby informing future vaccination initiatives. As of June 2023, 17.05 million individuals in the DRC had received at least one dose of COVID-19 vaccine [7]. To accelerate this effort, the Ministry of Public Health of the DRC, through the Expanded Programme on Immunization (EPI), and with support from Africa Centres for Disease Control and Prevention (Africa CDC) and partners, conducted a two-week Rapid Results Initiative (RRI) mass vaccination campaign. From December 1–29, 2023, in Kinshasa province, selected vaccine recipients were recruited for the follow-up monitoring. Alongside the campaign, Africa CDC and EPI carried out active safety surveillance to document AEFIs.

During the campaign, two COVID-19 vaccines were administered: the Pfizer-BioNTech mRNA vaccine (BNT162b2) and the Johnson & Johnson (J&J) non-replicating vector vaccine. The Pfizer-BioNTech vaccine employs mRNA technology to instruct the immune system to recognize the coronavirus spike protein [8]. In contrast, the J&J vaccine (Ad26.COV2.S) employs a recombinant adenovirus vector to deliver the gene for the spike protein into host cells [9].

## Methods

### Study design

An active COVID-19 vaccine safety surveillance was conducted through an observational prospective single-arm cohort study carried under the Ministry of Public Health of the DRC. Study participants were actively followed-up until 28 days after their COVID-19 vaccine.

### Participant selection and sampling

The COVID-19 mass vaccination campaign occurred in 159 vaccination sites distributed in 29 health zones of Kinshasa Province. In each vaccination site, according to the WHO recommendation, there was an intensive enrolment period from the start of the vaccination campaign until the predefined target number (study sample size) of enrolled individuals was reached [5]. The time point for enrolment was the vaccination with any licensed COVID-19 vaccine in one of the participating sites. Those participants who received COVID-19 vaccines during the campaign consented to participate in the study and provided a reachable phone number. Study recruitment was monitored during the study to assess whether recruitment goals are being reached.

### Sampling technique and sample size

Sample size was calculated, considering different event frequencies to guide the decision for suitable sample size. Based on the CIOMS guide to Active Vaccine Safety Surveillance, the target study size was 4766 individuals vaccinated with one or more dose(s) of COVID 19 vaccines [10].

If no event was observed, this study size could rule out events with a frequency of at least 1 per 1,588 with at least 95% confidence. The precision was defined as the half-width of the 95% exact confidence interval calculated using the Clopper-Pearson exact method [11]. Based on a sample size of 4766 participants, an anticipated prevalence of 10–15% could be estimated with 2% precision.

### Data collection and management

A structured questionnaire was adapted from the WHO COVID-19 Vaccine safety surveillance tool [5], and included predefined AEFI items aligned with WHO-recommended Adverse Events of Special Interest (AESI). It also included an open field for additional participant-reported AEFIs, which were coded prior to analysis. The tool captured data on population characteristics such as patient ID, province, health zone, sex, and age, as well as vaccine-related information such as batch number, number of doses, type of vaccine, vaccination date, and the record of AEFI. The data concerning AEFI included the date of the call, the date of onset of the event and the type of AEFI. Follow-up calls were made on days 1, 3, 5, 7, 14, 21, and 28 after vaccine administration and data collection forms were used to record information. The AEFI was considered serious in case of an event that results in death, hospitalization, or prolongation of an existing hospitalization, persistent or significant disability or incapacity, congenital anomaly/birth defect, or is life-threatening or is a medically important event or reaction [5]. For an appropriate follow up and care, any serious adverse event detected and reported in the context of this study was reported by the site to the MOH and the ethics committee within 24 hours of the site becoming aware. Fatal and life-threatening, serious adverse reactions had to be reported to the National Pharmacovigilance Centre within seven calendar days. Africa CDC recruited and trained a local team of AEFI focal points to conduct follow-up calls to identify and monitor the AEFI. A data collection line listing form with built-in validation rules was used to ensure data quality during data entry.

### Data analysis

#### Outcome variables.

The study’s primary outcome was the experience of at least one AEFI among those receiving at least one dose of any COVID-19 vaccine used during the vaccination mass campaign.

Eight Adverse Events of Special Interest (AESI), pre-defined for surveillance based on WHO recommendations— including fever, vomiting, headache, muscle pain, joint pain, body aches, persistent pain at the injection site, diarrhea, and chills—were actively monitored and assessed. We dichotomized the number of events as 2 or more coded as one (1) and 0–1 coded as zero (0) for our logistic regression model for the number of AEs.

#### Independent variables.

The study aimed at identifying factors associated with the incidence of AEFIs occurrence. The independent variables considered in this analysis included sex, type of COVID-19 vaccines, the number of COVID-19 vaccine doses received and age. During analysis, age was categorized into 3 age groups: < 18 years coded one (1), 18–59 coded zero (0) and ≥60 years coded two (2). This categorization of age based on the fact that extreme ages (<18 years and ≥60 years) have different type of immune system response compared to the remaining population [12,13].

#### Statistical analysis.

Descriptive statistics were used to summarize socio-demographic characteristics and vaccine-related information by frequency and proportion for categorical variables and median with interquartile range (IQR) for numerical variables. The cumulative incidence rate of AEFI was provided by proportion along with its respective 95% confidence interval. In bivariate analysis, the pearson chi-2 test was used to test association between categorical variables. In face of cells with values <5, Fisher’s exact test was used. The multivariable regression models using the backward-elimination regression approach included all the above-mentioned independent variables. Multicollinearity was assessed by the variance inflation factor (VIF) and the tolerance test (≥0.1). The VIF below ten was considered acceptable to declare lack or absence of multicollinearity. The goodness of model was assessed by the Hosmer Lemeshow goodness of fit test (P-value >0.05). Multivariable logistic regression models identified factors associated with experiencing AEFI and expressed by adjusted odds ratio (aOR) and its 95% CI. A p-value < 0.05 was used to declare statistical significance. Stata IC version 16 (Stata Corp) was used to perform this analysis.

### Ethical considerations

After the vaccine was administered, selected participants were informed about the purpose of the surveillance and invited to voluntarily take part in the survey. Verbal consent was obtained and documented on the follow-up form for each vaccine recipient, with the enumerator serving as a witness. Participants who consented provided their phone numbers for follow-up calls. For recipients under the age of 18, phone numbers of their parents or guardians were collected. To maintain confidentiality, only de-identified data were shared with the Africa CDC for analysis of the vaccine’s safety profile post-immunization. The Ministry of Public Health and the EPI program conducted the vaccination campaign and provided clearance for the subsequent vaccine safety surveillance. Ethical approval for conducting this survey and publishing the findings was obtained from the Ethics Committee of the School of Public Health at University of Kinshasa.

### Inclusivity in global research

Additional information regarding the ethical, cultural, and scientific considerations specific to inclusivity in global research is included in the Supporting Information (S1 Checklist).

## Results

### Population and vaccine characteristics

The analysis included 4766 participants, with a median age of 36 (Interquartile Range [IQR]: 27–48), min = 5 years, max = 90 years (S3 Table). More than half (53%) of the participants were males, and more than 70% of respondents were in the 20—to 49-year age range. Most participants (94.63%) received the Johnson & Johnson vaccine, and the remaining 5.37% were vaccinated with the BNT162b2 vaccine. Those taking their second dose were 0.57%, and 99.43% took their first dose (Table 1).

**Table 1:** Participants and vaccine characteristics.

Variables (N = 4766)	Group	N	% (95%CI)
**Sex**	Female	2,263	47.48% (46.06%−48.91%)
	Male	2,503	52.51% (51.09%−53.94%)
**Vaccine type**	J&J	4,510	94.63% (93.73%−95.05%)
	BNT162b2	256	5.37% (4.95%−6.27%)
**Number of doses**	First vaccination	4,739	99.43% (99.18%−99.63%)
	Second vaccination	27	0.57% (0.37%−0.82%)
**Age Groups**	<18 years	110	2.31% (1.90%−2.78%)
	18-59 years	4,189	87.89% (86.93%−88.81%)
	60 + years	467	9.79% (8.97%−10.68%)
**Seriousness of AEFIs**	Serious	6	0.13% (0.05%−0.27%)
	Non-serious	4,760	99.87% (97.73%−99.95%)

### Incidence and forms of AEFI reported

**Fig 1:**
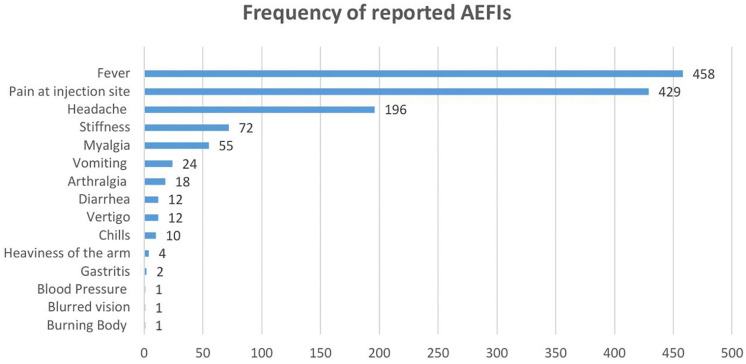
Frequency of reported AEFIs. This figure illustrates the types and number of AEFIs reported by the study participants.

Overall, 1132 participants (23.75%, 95% CI: 22.55% − 24.99%) reported experiencing at least one AEFI during the study period. The most commonly reported AEFIs, occurring in 1% to 10% of participants, were fever (458 cases; 9.61%, 95%CI: 8.88% −10.48%), pain at injection site (429 cases; 9.00%, 95%CI: 8.20% −9.85%), headache (196 cases; 4.11%, 95%CI: 3.57%−4.72%), stiffness (72 cases; 1.51%, 95%CI: 1,18% −1.89%) and myalgia (55 cases; 1.15%, 95%CI: 0.87% −1.49%). The less frequent reported AEFIs (ranging between 0.1% to 1%) included vomiting (24 cases; 0.50%, 95%CI: 0.32%−0.75%), arthralgia (18 cases; 0.38%, 95%CI: 0.22%−0.59%), diarrhea (12 cases; 0.25%, 95%CI: 0.13%−0.44%), vertigo (12 cases; 0.25%, 95%CI: 0.13%−0.44%) and chills (10 cases; 0.21%, 95%CI: 0.10%−0.38%). The unusual AEFIs, occurring at rates between 0.01% to 0.1%, included heaviness in the arm (4 cases; 0.08%, 95%CI: 0.02%−0.21%), gastritis (2 cases; 0.04%, 95%CI: 0.01%−15%), elevated blood pressure (1 case; 0.02%, 95%CI: 0.01%−0.11%)), blurred vision (1 case; 0.02%, 95%CI: 0.01%−0.11%) and a burning sensation (1 case; 0.02%, 95%CI: 0.01%−0.11%) (Fig 1)

**Fig 2:**
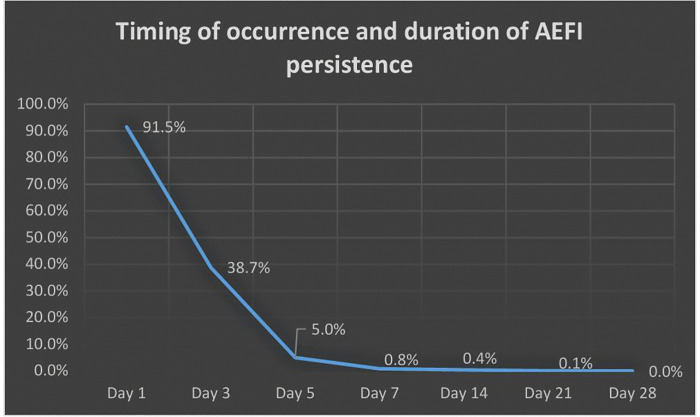
Timing of occurrence and duration of AEFI persistence. Fig 2 shows the decrease in percentage of COVID 19 vaccine recipients reporting any AEFI from day 1 onwards.

Of the 1,132 reported AIFIs, 1,035 (91.43%) occurred within the first 24 hours. By day 3, fewer than half—38.78% (436/1,132)—were still being reported, and only 5.02% of reactions persisted through day 5 (Fig 2).

**Fig 3:**
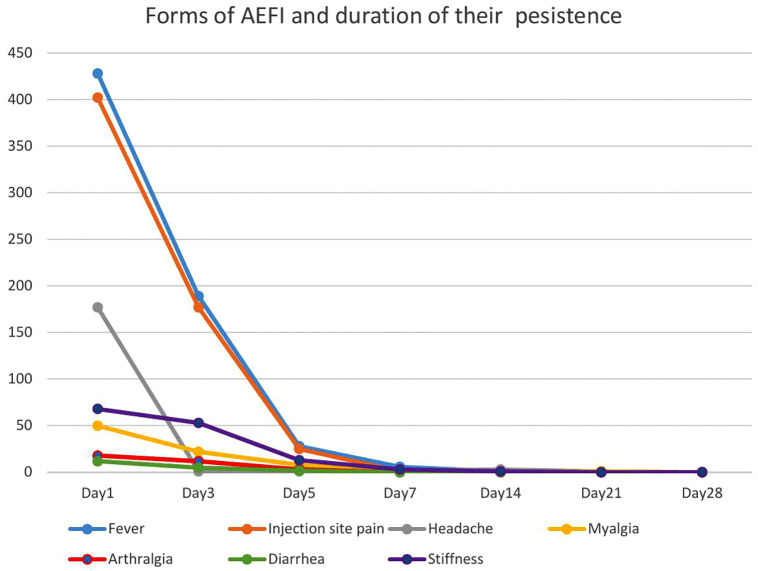
Forms of AEFI and duration of their persistence. Fig 3 disaggregates different forms of AEFIs and highlights the persistence of each throughout the follow up period.

Nearly all reported cases of headache had resolved within three days (Fig 3).

### Factors associated with the incidence of AEFIs

Incidence of AEFI was higher among BNT162b2 vaccine recipients (34.38%, 95% CI: 28.57%%−40.54%) versus J&J vaccine recipients (23.15%, 95% CI: 21.92%−24.41%); lower among vaccine recipients below 18 years of age (8.18%, 95%CI: 3.81%−14.96%) and those aged 60 years and older (20.34%, 95% CI: 16.78%−24.28%) versus those between 18–59 years of age (24.54%, 95% CI: 23.24%−25.97%). In the multivariable analysis, the occurrence of an AEFI was statistically associated with age and the type of vaccine administered. The odds of any AEs were about two times higher in the BNT162b2 vaccine (aOR, 1.79; 95% CI: 1.36–2.37); p < 0.001) as compared to J & J vaccine recipients. These odds were 74% lower among the younger age group (aOR, 0.26, 95%CI: 0.13–0.88; p < 0.001) at 17 years and younger as well as 22% lower among older age group (aOR, 0.78, 95%CI: 0.62–0.99; p < 0.044) at 60 years and more vs. the age group of 18–59 years. No statistical association was found for sex and number of doses (Table 2).

**Table 2:** Factors associated with the incidence of AEFIs.

Independent variables	Group	AEFI (-)	AEFI (+)	% AEFI	aOR (95%CI) ^a^	P-value
**Sex**	Male	1,908	595	23.77%	1.02 (0.89-1.16)	0.804
	Female	1,726	537	23.70%	1 (Ref)	
**Age**	<18 years	101	9	8.18%	0.26 (0.13 −0.88)	<0.001*
	≥60 years	372	95	20.34%	0.78 (0.62-0.99)	0.044*
	18-59 years	3,161	1,028	24.54%	1 (Ref)	
**Vaccine type**	BNT162b2	168	88	34.38%	1.79 (1.36-2.37)	<0.001*
	J & J Vaccine	3,466	1,044	23.15%	1 (Ref)	
**Number of doses**	1^st^ dose	3,612	1,126	23.77%	1.79 (0.69-4.61)	0.226
	2^nd^ dose	22	6	21.43%	1 (Ref)	

^a^ Multivariable Logistic regression; * Significant factors, p < 0.05.

Fever was lower for vaccine recipients aged 60 years and more (6.64%, 95%CI; 4.55%−9. 19%) than vaccine recipients aged 18−59 years (10.09%, 95%CI: 9.23%−11.05%). Vaccine recipients of 60 years of age and more were less likely susceptible to developing fever than recipients of the age group between 18 and 59 years (aOR=0.63, 95%CI: 0.43–0.92; p < 0.017). Respondents aged 17 years and younger had an 82% decreased risk of reporting pain at the site of injection compared to those aged 18−59 years (aOR=0.18, 95%CI: 0.05–0.75; p < 0.018) (Table 3).

**Table 3:** Different forms of AEFIs depending on age.

Side Effect	Age Group	AEFI (-)	AEFI (+)	% AEFI	aOR (95%CI) ^a^	P-value
**Fever**	<18 years	103	7	6.36%	0.59 (0.27-1.29)	0.194
	≥60 years	436	31	6.66%	0.63 (0.43-0.92)	0.017*
	18-59 years	3,769	420	10.09%	1 (Ref)	
**Injection site pain**	<18 years	108	2	1.82%	0.18 (0.05-0.75)	0.018*
	≥60 years	424	43	9.21%	1.03 (0.74-1.43)	0.871
	18-59 years	3,805	304	9.17%	1 (Ref)	
**Headache**	<18 years	109	1	0.91%	0.17 (0.02-1.29)	0.088
	≥60 years	452	15	3.21%	0.73 (0.42-1.26)	0.261
	18-59 years	4,009	180	4.30%	1 (Ref)	
**Vomiting**	<18 years	110	0	0.00%	omitted ^b^	
	≥60 years	466	1	0.21%	0.39 (0.05-2.88)	0.355
	18-59 years	4,166	23	0.55%	1 (Ref)	
**Muscle pain (Myalgia)**	<18 years	110	0	0.00%	omitted ^b^	
	≥60 years	460	7	1.50%	1.13 (0.59-2.92)	0.506
	18-59 years	4,141	48	1.15%	1 (Ref)	
**Joint pain (Arthralgia)**	<18 years	110	0	0.00%	omitted ^b^	
	≥60 years	465	2	0.43%	1.21 (0.26-4.89)	0.798
	18-59 years	4,173	16	0.38%	1 (Ref)	
**Diarrhea**	<18 years	110	0	0.00%	omitted ^b^	
	≥60 years	466	1	0.21%	0.81 (0.10-6.32)	0.851
	18-59 years	4,178	11	0.26%	1 (Ref)	
**Stiffness**	<18 years	109	1	0.91%	0.06 (0.08-4.37)	0.615
	≥60 years	459	8	1.71%	1.14 (0.54-2.39)	0.727
	18-59 years	4,126	63	1.50%	1 (Ref)	
**2 signes or more**	<18 years	109	1	0.91%	0.16 (0.02-1.20)	0.075
	≥60 years	448	19	4.07%	0.89 (0.54-1.44)	0.628
	18-59 years	3,998	191	4.56%	1 (Ref)	

^a^ Multivariable Logistic regression; * Significant factors, p < 0.05; ^b^ omitted from multivariable model due to zero cells.

Fever was higher for BNT162b2 vaccine recipients (12.50%, 95%CI; 8.71%−17.18%) than J&J vaccine recipients (9.45%, 95%CI: 4.35%−10.34%). Recipients of the BNT162b2 vaccine were more likely susceptible to developing fever than recipients of the J&J vaccine (aOR=1.50, 95%CI: 1,02–2.20; p < 0.041). The proportion of persistent pain at the injection site was higher for BNT162b2 vaccine recipients (15. 23%, 95%CI: 11.06%−20.23%%) vs J&J vaccine recipients (8.65%, 95%CI: 4.29%−9.51%). Respondents vaccinated with the BNT162b2 Vaccine had a 82% increased risk of reporting pain at the injection site than those vaccinated with the J&J vaccine (aOR=1.82,95%CI: 1.24–2.65; p < 0.002). Headache was more reported among BNT162b2 vaccine recipients (9.77%, 95%CI: 6.42%−14.07) than J&J vaccine recipients (3.79%, 95%CI: 3.25%−4.39%), muscle pain was reported by 3.52% (95%CI: 1.62%−6.57%) of BNT162b2 vaccine recipients vs 1.02% (95%CI; 0.75%−1.35) of J&J vaccine recipients. While all AEs were more predominant in BNT162b2 vaccine than in J&J vaccine, the odds were particularly higher for headache (aOR=2.92, 95%CI: 1.87–4.58; p < 0.001), myalgia (aOR=3.94, 95%CI: 1.90–8.15; p < 0.001) and reporting two signs or more (aOR=3.11, 95%CI: 2.03–4.77; p < 0.001) (Table 4).

**Table 4:** Different forms of AEFIs depending on the vaccine preparation.

Side Effect	Vaccine type	AEFI (-)	AEFI (+)	% AEFI	aOR (95%CI) ^a^	P-value
**Fever**	BNT162b2	224	32	12.50%	1.50 (1. 02-2.20)	0.041*
	J&J	4,084	426	9.45%	1 (Ref)	
**Injection site pain**	BNT162b2	217	39	15.23%	1.82 (1.24-2.65)	0.002*
	J&J	4,120	390	8.65%	1 (Ref)	
**Headache**	BNT162b2	231	25	9.77%	2.92 (1.87-4.58)	<0.001*
	J&J	4,339	171	3.79%	1 (Ref)	
**Vomiting**	BNT162b2	265	1	0.52%	0.48 (0.61-3.92)	0.478
	J&J	4,477	23	0.38%	1 (Ref)	
**Muscle pain (Myalgia)**	BNT162b2	247	9	3.52%	3.94 (1.90-8.15)	<0.001*
	J&J	4,464	46	1.02%	1 (Ref)	
**Joint pain (Arthralgia)**	BNT162b2	265	2	0.36%	1.14 (0.24-5.39)	0.869
	J&J	4,484	16	0.75%	1 (Ref)	
**Diarrhea**	BNT162b2	266	0	0.00%	Omitted ^b^	
	J&J	4,488	12	0.27%	1 (Ref)	
**Stiffness**	BNT162b2	253	3	1.17%	0.73 (0.23-2.34)	0.600
	J&J	4,441	69	1.53%	1 (Ref)	
**2 signes or more**	BNT162b2	228	28	10.94%	3.11 (2.03-4.77)	<0.001*
	J & J	4,327	183	4.06%	1 (Ref)	

^a^ Multivariable Logistic regression; * Significant factors, p < 0.05; ^b^ omitted from multivariable model due to zero cells.

## Discussion

Our study provides critical post-marketing safety data for COVID-19 vaccines from a mass vaccination campaign in the Democratic Republic of Congo, a population underrepresented in initial clinical trials. We found that approximately one-quarter of participants reported at least one adverse event following immunization (AEFI), with the incidence and type of AEFIs significantly associated with the vaccine preparation type and the recipient’s age.

### Incidence and comparison with other studies

The overall AEFI incidence of 23.75% in our study aligns with reports from clinical trials. For instance, the pivotal trial for the BNT162b2 mRNA vaccine reported any adverse event in 27% of vaccine recipients compared to 12% in the placebo group [14]. We observed a higher incidence of AEFIs with the BNT162b2 vaccine (34.38%) compared to the Johnson & Johnson vaccine (23.15%), a pattern consistent with the established reactogenicity profile of mRNA platforms [14,15].

However, our reported AEFI rates are considerably lower than those from intensive participant-reported surveys, such as a UK app-based study which found local side effects in 71.9% of participants after the first dose [16] and a US cohort where 80.3% of fully vaccinated individuals reported AEFIs [17]. This discrepancy may be attributed to several factors. The study was conducted in December 2023, by which time a significant proportion of the population had likely been previously infected with SARS-CoV-2. A non-primary immune response upon vaccination may have resulted in better tolerance and fewer reactogenic events. Furthermore, we did not assess the use of pre-medication, such as paracetamol, which was common in other settings like Ghana [18] and could attenuate AEFIs. Additionally, in active surveillance like ours, participants without AEFIs are systematically captured, whereas in voluntary reporting systems, individuals without symptoms may be less motivated to report, potentially inflating the apparent incidence.

### Spectrum of AEFIs

The most common AEFIs in our cohort were fever (9.61%), pain at injection site (9.00%), headache (4.11%), stiffness (1.51%) and myalgia (1.15%). This spectrum is consistent with global safety data for COVID-19 vaccines [15]. While direct comparisons are challenging due to differences in the predominant vaccines studied, the prevalence of local reactions like injection site pain as a top-tier event is a consistent finding across studies in Ethiopia [19] and Ghana [18]. Although not statistically significant in our cohort, we also observed a trend of increased injection site pain after the second dose (18.5% vs. 8.9% after the first), which corroborates findings from clinical trials where reactogenicity was often heightened following a second dose [14,20].

### Seriousness of AEFIs

Reassuringly, the incidence of serious adverse events was very low (0.13%), aligning with the low rate observed in the BNT162b2 clinical trial (0.6% in the vaccine group vs. 0.5% in placebo) [14].

### Factors associated with AEFIs

Multivariable analysis confirmed that vaccine type was the strongest factor associated with AEFIs. Recipients of the BNT162b2 (mRNA) vaccine had significantly greater odds of reporting headache (aOR=2.92), injection site pain (aOR=1.82), myalgia (aOR=3.94), and multiple symptoms (aOR=3.11) compared to recipients of the J&J vaccine. This finding is supported by a meta-analysis of clinical trials which concluded that mRNA-based vaccines were associated with the highest risk of systemic and local reactogenicity [15].

Age was also a significant factor. We found decreased odds of reporting any AEFI in both younger (<18 years) and older (≥60 years) participants compared to those aged 18–59. This is consistent with the known immunosenescence in older adults and the more robust immune responses typically seen in younger adults, as reported in studies from Japan [21] and other RCTs [12]. Our finding that participants under 20 were less likely to report AEFIs is further supported by Abukhalil et al. [13].

Contrary to many other studies [17,22], we did not find a significant association between female sex and higher AEFI incidence. This divergence merits further investigation. It is noteworthy that other studies have reported menstrual disorders and unexpected vaginal bleeding post-vaccination in females, which were not specifically monitored in our surveillance. These events warrant careful monitoring in future studies.

### Active surveillance as a complement to spontaneous reporting

Spontaneous reporting systems, by their passive design, have a limited capacity to detect mild adverse events following immunization, as exemplified in the DRC [5]. Our study addressed this gap by employing active surveillance to generate essential insights into COVID-19 vaccine safety, complementing the routine national pharmacovigilance data. The active surveillance approach mirrors validated models like cohort event monitoring for antimalarials and other medications [23,24]. The results reinforce the CIOMS recommendation that comprehensive post-marketing safety evaluation necessitates supplementing passive reports with systematic active monitoring [10]. The study provided crucial population-specific data, showing lower AEFI odds in the very young and old. This is vital for building a complete safety profile and public trust, especially in regions underrepresented in clinical trials. The strongly decreased odds of AEFIs in those under 18 and over 60 provides reassuring local safety data for vulnerable age groups, supporting continued vaccination efforts for the elderly and paving the way for pediatric vaccination campaigns. This study serves as a proof-of-concept for implementing advanced pharmacovigilance in a low-resource setting, a valuable methodological contribution for global health security.

A telephone-based system offers advantages in scalability, cost-effectiveness, and geographical reach, facilitating follow-up of large, dispersed populations. The convenience and perceived anonymity may also improve reporting rates. However, key limitations include the inability to conduct physical examinations, potential for lower engagement or truncated responses, and selection bias against those without reliable phone access. This method can be effectively integrated into community pharmacy vaccination programs. A multi-modal strategy combining scheduled calls, SMS check-ins, and a dedicated reporting line could establish an inclusive, low-cost channel for community-based pharmacovigilance.

### Limitations

Our results should be interpreted with caution given the following limitations. First, there might be potential misclassification of outcomes, as AEFIs reported by the participants were not medically reviewed and classification of the AEFI has not been conducted. Secondly, the low number of second-dose recipients and absence of heterologous vaccination limited our ability to conduct meaningful subgroup analyses. Future or repeated active surveillance rounds will be required to assess second-dose and heterologous vaccination safety. Thirdly, as we only examined the short-term safety of COVID-19 vaccines, further studies are required to evaluate the long-term AEs. Lastly, this survey did not assess for any pre-existing medical conditions or whether participants were pre-medicated before vaccination. The absence of paracetamol use assessment is a limitation for this survey.

The study might also have potential biases. Exclusion of individuals without phone access could constitute a selection bias, affecting representativeness. The telephone-based recruitment and follow-up approach may have introduced selection bias and reduced representativeness among younger and older age groups (<18 years and ≥60 years). The broad age grouping (18–59 years) may have masked differences between younger and middle-aged adults and should be explored in future studies with larger samples.

## Conclusion

In conclusion, this survey conducted in Kinshasa (DRC) revealed that approximately 24% of COVID-19 vaccine recipients aged 5 − 90 years reported AEFIs, and most of AEFIs were mild and transient. Fever, Injection site pain, headache, and muscle pain were the most predominant AEFIs reported by COVID-19 vaccine recipients. We also found that vaccination with the BNT162b2 vaccine and age of 18–59 years were associated with the incidence of AEFIs. The BNT162b2 vaccine was significantly associated with greater odds of reporting fever, headache, muscle pain, injection site pain, and more than 2 signs compared to the J&J vaccine. Individuals aged 60 years or more and 17 years or less were associated with lower odds of reporting AEFI. Further safety surveys monitoring all potential factors are needed to generate more evidence on COVID-19 vaccine safety in an African population. This information will support establishing future vaccination strategies tailored to individuals potentially susceptible to AEFIs.

## Supporting information

S1 Checklist Inclusivity in global research. (DOCX)

S1 Table Factors associated with different forms of AEFIs. (DOCX)

S2 Table Factors associated with the number of signs reported by an individual. (DOCX)

S3 Table Mean and median age. (DOCX)

S4 Table Data collection tool (Questionnaire). (DOCX)

S5 Table De-identified dataset. (XLSX)

S1 Fig Age distribution of the study participants. (DOCX)

S6 Table Distribution of less frequent AEFIs by age sex and vaccine type. (DOCX)
