# Supplementary material for: Adverse events following immunization during COVID-19 mass vaccination campaigns in the Democratic Republic of Congo: Findings from active safety surveillance
Source: PLoS One. 2026 Jul 10;21(7):e0309628. doi: 10.1371/journal.pone.0309628 (PMC13353984; doi:10.1371/journal.pone.0309628)
Supplement: S1 Table — (DOCX) [file pone.0309628.s002.docx]

***A. Factors associated with Fever***

| **Independent Variables** | **Group** | **Fever(-)** | **Fever (+)** | **% Fever** | **aOR (95%CI)** ^a^ | **P-value** |
| --- | --- | --- | --- | --- | --- | --- |
| Sex | Male | 2,255 | 248 | 9.91% | 1.07 (0.89-1.30) | 0.452 |
|  | Female | 2,053 | 210 | 9.28% | 1 (Ref) |  |
| Age | <18 years | 103 | 7 | 6.36% | 0.59 (0.27-1.29) | 0.194 |
|  | ≥60 years | 436 | 31 | 6. 66% | 0. 63 (0.43-0.92) | 0.017* |
|  | 18-59 years | 3,769 | 420 | 10.09% | 1 (Ref) |  |
| Type of Vaccine | BNT162b2 | 224 | 32 | 12.5% | 1.50 (1. 02-2.20) | 0.041* |
|  | J & J Vaccine | 4,084 | 426 | 9.45% | 1 (Ref) |  |

^a^ Multivariable Logistic regression; * Significant factors, p<0.05

***B. Factors associated with persistent pain at the injection site***

| **Independent variables** | **Groups** | **Pain (-)** | **Pain (+)** | **% Injection site pain** | **aOR (95%CI)** ^a^ | **P-Value** |
| --- | --- | --- | --- | --- | --- | --- |
| Sex | Male | 2,280 | 223 | 8.91% | 0.97(0.79-1.18) | 0.756 |
|  | Female | 2,057 | 206 | 9.10% | 1 (Ref) |  |
| Age group | <18 years | 108 | 2 | 1.82% | 0.18 (0.05-0.75) | 0.018* |
|  | ≥60 years | 424 | 43 | 9.21% | 1.03(0.74-1.43) | 0.871 |
|  | 18-59 years | 3,805 | 304 | 9.17% | 1 (Ref) |  |
| Type of Vaccine | BNT162b2 | 217 | 39 | 15.23% | 1.82(1.24-2.65) | 0.002* |
|  | J & J Vaccine | 4,120 | 390 | 8.65% | 1 (Ref) |  |
| Number of doses administered | 1^st^ dose | 4,315 | 424 | 8.95% | 0.75(0.27-2.12) | 0.598 |
|  | 2^nd^ dose | 22 | 5 | 18.52% | 1 (Ref) |  |

^a^ Multivariable Logistic regression; * Significant factors, p<0.05

***C. Factors associated with headache***

| **Independent Variables** | **Groups** | **Headache (-)** | **Headache (+)** | | **% Headache** | **OR (95%CI)** ^a^ | **P-Value** |
| --- | --- | --- | --- | --- | --- | --- | --- |
| Sex | Male | 2,398 | | 105 | 4.19% | 1.05 (0.79-1,40) | 0.707 |
|  | Female | 2,172 | | 91 | 4.02% | 1 (Ref) |  |
| Age | <18 years | 109 | | 1 | 0.91% | 0.17 (0.02-1.29) | 0.088 |
|  | ≥60 years | 452 | | 15 | 3.21% | 0.73 (0.42-1.26) | 0.261 |
|  | 18-59 years | 4,009 | | 180 | 4.30% | 1 (Ref) |  |
| Type of Vaccine | BNT162b2 | 231 | | 25 | 9.77% | 2.92 (1.87-4.58) | 0.001* |
|  | J & J Vaccine | 4,339 | | 171 | 3.79% | 1 (Ref) |  |
| Number of doses | 1^st^ dose | 4,544 | | 190 | 4.11% | 2.85(0.37-21.93) | 0.313 |
|  | 2^nd^ dose | 21 | | 6 | 3.70% | 1 (Ref) |  |

^a^ Multivariable Logistic regression; * Significant factors, p<0.05

***D. Factors associated with muscle pain***

| **Independent Variables** | **Groups** | **Myalgia (-)** | **Myalgia (+)** | **% Myalgia** | **aOR(95%CI)** ^a^ | **P-Value** |
| --- | --- | --- | --- | --- | --- | --- |
| Sex | Male | 2,474 | 29 | 1.16% | 1.01(0.59-1.73) | 0.96 |
|  | Female | 2,237 | 26 | 1.15% | 1 (Ref) |  |
| Age | <18 years | 110 | 0 | 0.00% | omitted ^b^ |  |
|  | ≥60 years | 460 | 7 | 1.50% | 1.13 (0.59-2.92) | 0.506 |
|  | 18-59 years | 4,141 | 48 | 1.15% | 1 (Ref) |  |
| Type of Vaccine | BNT162b2 | 247 | 9 | 3.52% | 3.94 (1.90-8.15) | 0.001* |
|  | J & J Vaccine | 4,464 | 46 | 1.02% | 1 (Ref) |  |
| Number of doses | 1^st^ dose | 4,684 | 55 | 1.16% |  |  |
|  | 2^nd^ dose | 27 | 0 | 0.00% | Omitted ^b^ |  |

^a^ Multivariable Logistic regression; * Significant factors, p<0.05; ^b^ Omitted from from the model due to zero cells

**Less frequent AEFIs**

***E. Vertigo***

| **Independent Variables** | **Group** | **Vertigo**  **(-)** | **Vertigo**  **(+)** | **% Vertigo** | **P-value** ^a^ |
| --- | --- | --- | --- | --- | --- |
| Sex | Male | 2,497 | 6 | 0.24% | 0.861 |
|  | Female | 2,257 | 6 | 0.27% |  |
| Age | <18 years | 110 | 0 | 0.00% | 1.000 |
|  | ≥60 years | 466 | 1 | 0.21% |  |
|  | 18-59 years | 4,178 | 11 | 0.26% |  |
| Type of Vaccine | BNT162b2 | 253 | 3 | 1.17% | 0.023 |
|  | J & J Vaccine | 4,501 | 9 | 0. 20% |  |
| Number of doses | 1^st^ dose | 4,726 | 12 | 0.25% | 0.952 |
|  | 2^nd^ dose | 28 | 0 | 0.00% |  |

^a^ Chi 2 test/Fisher’s exact test

***F. Chills***

| **Independent Variables** | **Group** | **Chills (-)** | **Chills (+)** | **% Chills** | **P-value** ^a^ |
| --- | --- | --- | --- | --- | --- |
| Sex | Male | 2,501 | 2 | 0.08 % | 0.055 |
|  | Female | 2,255 | 8 | 0.35% |  |
| Age | <18 years | 110 | 0 | 0.00% | 0.693 |
|  | ≥60 years | 467 | 0 | 0.00% |  |
|  | 18-59 years | 4,179 | 10 | 0.24% |  |
| Type of Vaccine | BNT162b2 | 255 | 1 | 0.39% | 0.425 |
|  | J & J Vaccine | 4,501 | 9 | 0.20% |  |
| Number of doses | 1^st^ dose | 4,728 | 10 | 0.21% | 0.943 |
|  | 2^nd^ dose | 28 | 0 | 0.00% |  |

^a^ Fisher’s exact test

***G. Physical asthenia***

| **Independent Variables** | **Group** | **Physical asthenia**  **(-)** | **Physical asthenia**  **(+)** | **% Physical asthenia** | **P-value** ^a^ |
| --- | --- | --- | --- | --- | --- |
| Sex | Male | 2,501 | 2 | 0.08% | 0.920 |
|  | Female | 2,261 | 2 | 0.09% |  |
| Age | <18 years | 110 | 0 | 0.00% | 1.000 |
|  | ≥60 years | 467 | 0 | 0.00% |  |
|  | 18-59 years | 4,185 | 4 | 0.10% |  |
| Type of Vaccine | BNT162b2 | 256 | 0 | 12.5% | 0.802 |
|  | J & J Vaccine | 4,506 | 4 | 9.45% |  |
| Number of doses | 1^st^ dose | 4,734 | 4 | 0.08% | 0.977 |
|  | 2^nd^ dose | 28 | 0 | 0.00% |  |

^a^ Fisher’s exact test

1. ***Heaviness of the arm***

| **Independent Variables** | **Group** | **Heaviness**  **(-)** | **Heaviness (+)** | **% Heaviness** | **P-value** ^a^ |
| --- | --- | --- | --- | --- | --- |
| Sex | Male | 2,501 | 2 | 0.08% | 0.649 |
|  | Female | 2,261 | 2 | 0.09% |  |
| Age | <18 years | 110 | 0 | 0.00% | 1.000 |
|  | ≥60 years | 467 | 0 | 0.00% |  |
|  | 18-59 years | 4,185 | 4 | 0.08% |  |
| Type of Vaccine | BNT162b2 | 256 | 0 | 0.00% | 0.802 |
|  | J & J Vaccine | 4,185 | 4 | 0.09% |  |
| Number of doses | 1^st^ dose | 4,734 | 4 | 0.08% | 0.977 |
|  | 2^nd^ dose | 28 | 0 | 0.00% |  |

^a^ Fisher’s exact test

1. ***Increased Blood Pressure***

| **Independent Variables** | **Group** | **Increased BP**  **(-)** | **Increased BP**  **(+)** | **% Increased BP** | **P-value** ^a^ |
| --- | --- | --- | --- | --- | --- |
| Sex | Male | 2,502 | 1 | 0.04% | 1.000 |
|  | Female | 2,263 | 0 | 0.00% |  |
| Age | <18 years | 110 | 0 | 0.00% | 1.000 |
|  | ≥60 years | 467 | 0 | 0.00% |  |
|  | 18-59 Years | 4,188 | 1 | 0.02% |  |
| Type of Vaccine | BNT162b2 | 256 | 0 | 0.00% | 1.000 |
|  | J & J Vaccine | 4,509 | 1 | 0.02% |  |
| Number of doses | 1^st^ dose | 4,765 | 1 | 0.02% | 0.994 |
|  | 2^nd^ dose | 28 | 0 | 0.00% |  |

^a^ Fisher’s exact test

1. ***Blurred vision***

| **Independent Variables** | **Group** | **Blurred vision**  **(-)** | **Blurred vision**  **(+)** | **% Blurred vision** | **P-value** ^a^ |
| --- | --- | --- | --- | --- | --- |
| Sex | Male | 2,503 | 0 | 0.00% | 0.475 |
|  | Female | 2,262 | 1 | 0.04% |  |
| Age | <18 years | 110 | 0 | 0.00% | 0.121 |
|  | ≥60 years | 466 | 1 | 0. 21% |  |
|  | 18-59 years | 4,189 | 0 | 0.00% |  |
| Type of Vaccine | BNT162b2 | 256 | 0 | 0.00% | 1.000 |
|  | J & J Vaccine | 4,509 | 1 | 0.02% |  |
| Number of doses | 1^st^ dose | 4,737 | 1 | 0.02% | 0.994 |
|  | 2^nd^ dose | 28 | 0 | 0.00% |  |

^a^ Fisher’s exact test

1. ***Burning body sensation***

| **Independent Variables** | **Group** | **Burning boby (-)** | **Burning body (+)** | **% Burning body** | **P-value** ^a^ |
| --- | --- | --- | --- | --- | --- |
| Sex | Male | 2,503 | 0 | 0.00% | 0.475 |
|  | Female | 2,262 | 1 | 0.04% |  |
| Age | <18 years | 110 | 0 | 0.00% | 1.000 |
|  | ≥60 years | 467 | 0 | 0. 00% |  |
|  | 18-59 years | 4,188 | 1 | 0.02% |  |
| Type of Vaccine | BNT162b2 | 256 | 0 | 0.00% | 1.000 |
|  | J & J Vaccine | 4,509 | 1 | 0.02% |  |
| Number of doses | 1^st^ dose | 4,737 | 1 | 0.02% | 0.994 |
|  | 2^nd^ dose | 28 | 0 | 0.00% |  |

^a^ Fisher’s exact test

1. ***Gastritis***

| **Independent Variables** | **Group** | **Gastritis**  **(-)** | **Gastritis (+)** | **% Gastritis** | **P-value** ^a^ |
| --- | --- | --- | --- | --- | --- |
| Sex | Male | 2,503 | 0 | 0.00% | 0.225 |
|  | Female | 2,261 | 2 | 0.09% |  |
| Age | <18 years | 110 | 0 | 0.00% | 1.000 |
|  | ≥60 years | 467 | 0 | 0. 00% |  |
|  | 18-59 years | 4,187 | 2 | 0.09% |  |
| Type of Vaccine | BNT162b2 | 256 | 0 | 0.00% | 1.000 |
|  | J & J Vaccine | 4,508 | 2 | 0.04% |  |
| Number of doses | 1^st^ dose | 4,764 | 2 | 0.04% | 0.988 |
|  | 2^nd^ dose | 28 | 0 | 0.00% |  |

^a^ Fisher’s exact test
