# Supplementary material for: Adverse events following immunization during COVID-19 mass vaccination campaigns in the Democratic Republic of Congo: Findings from active safety surveillance
Source: PLoS One. 2026 Jul 10;21(7):e0309628. doi: 10.1371/journal.pone.0309628 (PMC13353984; doi:10.1371/journal.pone.0309628)
Supplement: S2 Table — (DOCX) [file pone.0309628.s003.docx]

| **Independent Variables** | **Groups** | **< 2 signs** | **2 signs and +** | **% 2 signs and +** | **aOR(95%CI)** | **P-value** |
| --- | --- | --- | --- | --- | --- | --- |
| Sex | Female | 2,163 | 100 | 4.43% | 0.99 (0.76-1.32) | 0.992 |
|  | Male | 2,392 | 111 | 4.42% | 1 (Ref) |  |
| Age | <18 years | 109 | 1 | 0.91% | 0.16 (0.02-1.20) | 0.075 |
|  | 60 Years and + | 448 | 19 | 4.07% | 0.89 (0.54-1.44) | 0.628 |
|  | 18-59 Years | 3998 | 191 | 4.56% | 1 (Ref) |  |
| Type of Vaccine | BNT162b2 | 228 | 28 | 10.94% | 3.11 (2.03-4.77) | 0.001* |
|  | J & J Vaccine | 4,327 | 183 | 4.06% | 1 (Ref) |  |
| Number of doses | 1^st^ dose | 4,529 | 211 | 4.43% | 3.37 (0.44-25.83) | 0.242 |
|  | 2^nd^ dose | 26 | 1 | 3.57% | 1 (Ref) |  |

***S2 Table. Factors associated with the number of signs reported by an individual***
