# Supplementary figures and images for: Adverse events following immunization during COVID-19 mass vaccination campaigns in the Democratic Republic of Congo: Findings from active safety surveillance
Source: PLoS One. 2026 Jul 10;21(7):e0309628. doi: 10.1371/journal.pone.0309628 (PMC13353984; doi:10.1371/journal.pone.0309628)

S 3 Table. Mean and Median Age
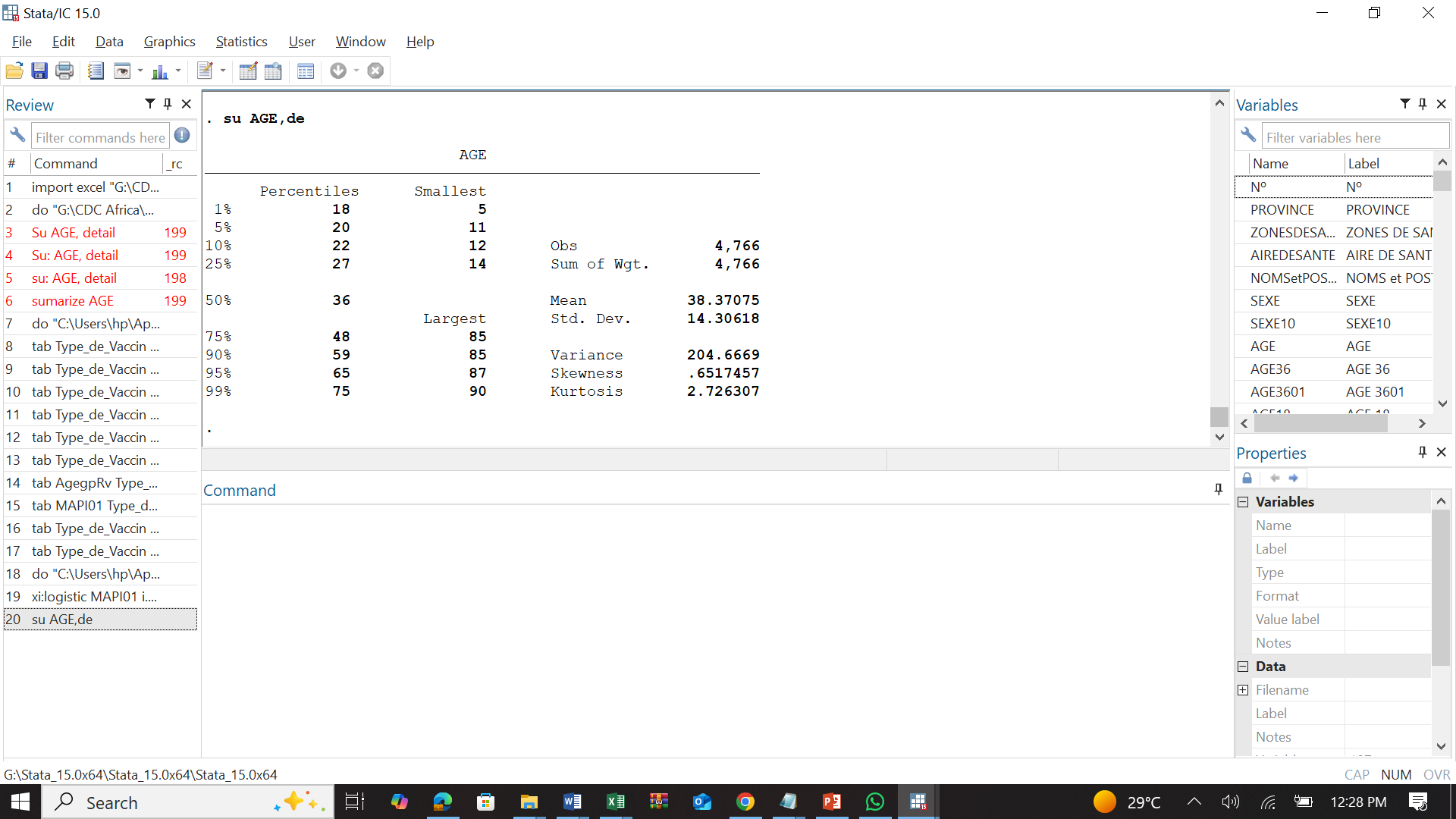

Supplement: S3 Table — (DOCX) [file pone.0309628.s004.docx]
