# Supplementary material for: Adverse events following immunization during COVID-19 mass vaccination campaigns in the Democratic Republic of Congo: Findings from active safety surveillance
Source: PLoS One. 2026 Jul 10;21(7):e0309628. doi: 10.1371/journal.pone.0309628 (PMC13353984; doi:10.1371/journal.pone.0309628)
Supplement: S4 Table — (DOCX) [file pone.0309628.s005.docx]

**VACCINATION AGAINST COVID-19 IN DRC, 2023**

**VACCINATED PERSONS MONITORING FORM (Active AEFI Surveillance)**

| Province : | Period of activity: | Team Supervisor (Name & Post name) : |
| --- | --- | --- |
| Health Zone : | Date (mm/dd/yyyy) : | Focal Point (Name & Post name) : |
| Health Area : | Health Centre : |  |
| Vaccination Site : | Village/Street/Avenue/ : | |

**N.B : For any person to be interviewed, verbal consent must be obtained, while explaining that the investigation is confidential**

**Clinical signs to look for:** No Complaints=0, Fever=1, Vomiting=2, Headache=3, Myalgia=4, Arthralgia=5, Stiffness=6, Persistent pain at the injection site =7, diarrhea=8, Others (to be specified on comment) =99. **Not reachable =**NJ, **Refusal to Respond**=RR,

| **N°** | **Names and Post name ( of vaccinee)** | **SEX** | **AGE Years** | **Pregnant ?/**  **Lactating ?** | **Vaccination Date Antigen received (dose 1 ou 2)** | **Batch Number/MAH** | **Téléphone Number** | **Seious AEFI ?(Y/N)**  **If Y, reason** | **Signs and date** | **J1** | **J3** | **J5** | **J7** | **14** | **J21** | **J28** | **COMMENT** |
| --- | --- | --- | --- | --- | --- | --- | --- | --- | --- | --- | --- | --- | --- | --- | --- | --- | --- |
| 1 |  |  |  |  |  |  |  |  | Signs |  |  |  |  |  |  |  |  |
|  |  |  |  |  |  |  |  |  | Date |  |  |  |  |  |  |  |  |
| 2 |  |  |  |  |  |  |  |  | Signs |  |  |  |  |  |  |  |  |
|  |  |  |  |  |  |  |  |  | Date |  |  |  |  |  |  |  |  |
| 3 |  |  |  |  |  |  |  |  | Signs |  |  |  |  |  |  |  |  |
|  |  |  |  |  |  |  |  |  | Date |  |  |  |  |  |  |  |  |
| 4 |  |  |  |  |  |  |  |  | Signs |  |  |  |  |  |  |  |  |
|  |  |  |  |  |  |  |  |  | Date |  |  |  |  |  |  |  |  |
| 5 |  |  |  |  |  |  |  |  | Signs |  |  |  |  |  |  |  |  |
|  |  |  |  |  |  |  |  |  | Date |  |  |  |  |  |  |  |  |
| 6 |  |  |  |  |  |  |  |  | Signs |  |  |  |  |  |  |  |  |
|  |  |  |  |  |  |  |  |  | Date |  |  |  |  |  |  |  |  |
| 7 |  |  |  |  |  |  |  |  | Signs |  |  |  |  |  |  |  |  |
|  |  |  |  |  |  |  |  |  | Date |  |  |  |  |  |  |  |  |
| 8 |  |  |  |  |  |  |  |  | Signs |  |  |  |  |  |  |  |  |
|  |  |  |  |  |  |  |  |  | Date |  |  |  |  |  |  |  |  |
| 9 |  |  |  |  |  |  |  |  | Signs |  |  |  |  |  |  |  |  |
|  |  |  |  |  |  |  |  |  | Date |  |  |  |  |  |  |  |  |
| 10 |  |  |  |  |  |  |  |  | Signs |  |  |  |  |  |  |  |  |
|  |  |  |  |  |  |  |  |  | Date |  |  |  |  |  |  |  |  |

**Sex :** Male=M, Female=F

**Vaccin (Antigen) and dose received :** PFIZER1=PFIZER first dose, PFIZER2=PFIZER second dose, (Idem for Johson-johson, Astrazeneca, …)
