# Supplementary material for: Adverse events following immunization during COVID-19 mass vaccination campaigns in the Democratic Republic of Congo: Findings from active safety surveillance
Source: PLoS One. 2026 Jul 10;21(7):e0309628. doi: 10.1371/journal.pone.0309628 (PMC13353984; doi:10.1371/journal.pone.0309628)
Supplement: S6 Table — (DOCX) [file pone.0309628.s008.docx]

**Distribution of less frequent AEFIs by age Sex and Vaccine type**

1. **Vertigo**

| **Independent Variables** | **Group** | **Vertigo**  **(-)** | **Vertigo**  **(+)** | **% Vertigo** | **P-value** ^a^ |
| --- | --- | --- | --- | --- | --- |
| Sex | Male | 2,497 | 6 | 0.24% | 0.861 |
|  | Female | 2,257 | 6 | 0.27% |  |
| Age | <18 years | 110 | 0 | 0.00% | 1.000 |
|  | 60 Years and + | 466 | 1 | 0.21% |  |
|  | 18-59 Years | 4,178 | 11 | 0.26% |  |
| Type of Vaccine | BNT162b2 | 253 | 3 | 1.17% | 0.023 |
|  | J & J Vaccine | 4,501 | 9 | 0. 20% |  |
| Number of doses | 1^st^ dose | 4,726 | 12 | 0.25% | 0.952 |
|  | 2^nd^ dose | 28 | 0 | 0.00% |  |

^a^ Chi 2 test/Fisher’s exact test

1. **Chills**

| **Independent Variables** | **Group** | **Chills (-)** | **Chills (+)** | **% Chills** | **P-value** ^a^ |
| --- | --- | --- | --- | --- | --- |
| Sex | Male | 2,501 | 2 | 0.08 % | 0.055 |
|  | Female | 2,255 | 8 | 0.35% |  |
| Age | <18 years | 110 | 0 | 0.00% | 0.693 |
|  | 60 Years and + | 467 | 0 | 0.00% |  |
|  | 18-59 Years | 4,179 | 10 | 0.24% |  |
| Type of Vaccine | BNT162b2 | 255 | 1 | 0.39% | 0.425 |
|  | J & J Vaccine | 4,501 | 9 | 0.20% |  |
| Number of doses | 1^st^ dose | 4,728 | 10 | 0.21% | 0.943 |
|  | 2^nd^ dose | 28 | 0 | 0.00% |  |

^a^ Fisher’s exact test

1. **Physical asthenia**

| **Independent Variables** | **Group** | **Physical asthenia**  **(-)** | **Physical asthenia**  **(+)** | **% Physical asthenia** | **P-value** ^a^ |
| --- | --- | --- | --- | --- | --- |
| Sex | Male | 2,501 | 2 | 0.08% | 0.920 |
|  | Female | 2,261 | 2 | 0.09% |  |
| Age | <18 years | 110 | 0 | 0.00% | 1.000 |
|  | 60 Years and + | 467 | 0 | 0.00% |  |
|  | 18-59 Years | 4,185 | 4 | 0.10% |  |
| Type of Vaccine | BNT162b2 | 256 | 0 | 12.5% | 0.802 |
|  | J & J Vaccine | 4,506 | 4 | 9.45% |  |
| Number of doses | 1^st^ dose | 4,734 | 4 | 0.08% | 0.977 |
|  | 2^nd^ dose | 28 | 0 | 0.00% |  |

^a^ Fisher’s exact test

1. **Heaviness of the arm**

| **Independent Variables** | **Group** | **Heaviness**  **(-)** | **Heaviness (+)** | **% Heaviness** | **P-value** ^a^ |
| --- | --- | --- | --- | --- | --- |
| Sex | Male | 2,501 | 2 | 0.08% | 0.649 |
|  | Female | 2,261 | 2 | 0.09% |  |
| Age | <18 years | 110 | 0 | 0.00% | 1.000 |
|  | 60 Years and + | 467 | 0 | 0.00% |  |
|  | 18-59 Years | 4,185 | 4 | 0.08% |  |
| Type of Vaccine | BNT162b2 | 256 | 0 | 0.00% | 0.802 |
|  | J & J Vaccine | 4,185 | 4 | 0.09% |  |
| Number of doses | 1^st^ dose | 4,734 | 4 | 0.08% | 0.977 |
|  | 2^nd^ dose | 28 | 0 | 0.00% |  |

^a^ Fisher’s exact test

1. **Increased Blood Pressure**

| **Independent Variables** | **Group** | **Increased BP**  **(-)** | **Increased BP**  **(+)** | **% Increased BP** | **P-value** ^a^ |
| --- | --- | --- | --- | --- | --- |
| Sex | Male | 2,502 | 1 | 0.04% | 1.000 |
|  | Female | 2,263 | 0 | 0.00% |  |
| Age | <18 years | 110 | 0 | 0.00% | 1.000 |
|  | 60 Years and + | 467 | 0 | 0.00% |  |
|  | 18-59 Years | 4,188 | 1 | 0.02% |  |
| Type of Vaccine | BNT162b2 | 256 | 0 | 0.00% | 1.000 |
|  | J & J Vaccine | 4,509 | 1 | 0.02% |  |
| Number of doses | 1^st^ dose | 4,765 | 1 | 0.02% | 0.994 |
|  | 2^nd^ dose | 28 | 0 | 0.00% |  |

^a^ Fisher’s exact test

1. **Blurred vision**

| **Independent Variables** | **Group** | **Blurred vision**  **(-)** | **Blurred vision**  **(+)** | **% Blurred vision** | **P-value** ^a^ |
| --- | --- | --- | --- | --- | --- |
| Sex | Male | 2,503 | 0 | 0.00% | 0.475 |
|  | Female | 2,262 | 1 | 0.04% |  |
| Age | <18 years | 110 | 0 | 0.00% | 0.121 |
|  | 60 Years and + | 466 | 1 | 0. 21% |  |
|  | 18-59 Years | 4,189 | 0 | 0.00% |  |
| Type of Vaccine | BNT162b2 | 256 | 0 | 0.00% | 1.000 |
|  | J & J Vaccine | 4,509 | 1 | 0.02% |  |
| Number of doses | 1^st^ dose | 4,737 | 1 | 0.02% | 0.994 |
|  | 2^nd^ dose | 28 | 0 | 0.00% |  |

^a^ Fisher’s exact test

1. **Burning body sensation**

| **Independent Variables** | **Group** | **Burning boby (-)** | **Burning body (+)** | **% Burning body** | **P-value** ^a^ |
| --- | --- | --- | --- | --- | --- |
| Sex | Male | 2,503 | 0 | 0.00% | 0.475 |
|  | Female | 2,262 | 1 | 0.04% |  |
| Age | <18 years | 110 | 0 | 0.00% | 1.000 |
|  | 60 Years and + | 467 | 0 | 0. 00% |  |
|  | 18-59 Years | 4,188 | 1 | 0.02% |  |
| Type of Vaccine | BNT162b2 | 256 | 0 | 0.00% | 1.000 |
|  | J & J Vaccine | 4,509 | 1 | 0.02% |  |
| Number of doses | 1^st^ dose | 4,737 | 1 | 0.02% | 0.994 |
|  | 2^nd^ dose | 28 | 0 | 0.00% |  |

^a^ Fisher’s exact test

1. **Gastritis**

| **Independent Variables** | **Group** | **Gastritis**  **(-)** | **Gastritis (+)** | **% Gastritis** | **P-value** ^a^ |
| --- | --- | --- | --- | --- | --- |
| Sex | Male | 2,503 | 0 | 0.00% | 0.225 |
|  | Female | 2,261 | 2 | 0.09% |  |
| Age | <18 years | 110 | 0 | 0.00% | 1.000 |
|  | 60 Years and + | 467 | 0 | 0. 00% |  |
|  | 18-59 Years | 4,187 | 2 | 0.09% |  |
| Type of Vaccine | BNT162b2 | 256 | 0 | 0.00% | 1.000 |
|  | J & J Vaccine | 4,508 | 2 | 0.04% |  |
| Number of doses | 1^st^ dose | 4,764 | 2 | 0.04% | 0.988 |
|  | 2^nd^ dose | 28 | 0 | 0.00% |  |

^a^ Fisher’s exact test
