# Supplementary material for: Adverse events following immunization during COVID-19 mass vaccination campaigns in the Democratic Republic of Congo: Findings from active safety surveillance
Source: PLoS One. 2026 Jul 10;21(7):e0309628. doi: 10.1371/journal.pone.0309628 (PMC13353984; doi:10.1371/journal.pone.0309628)
Supplement: Attachment — Submitted filename: Responses to Reviewers comments.pdf [file pone.0309628.s012.pdf]

October 27, 2025

Dear Nuru Abdu

Enclosed is a revised version of our manuscript, titled: “Adverse events following immunization during COVID-19 mass vaccination campaigns in the Democratic Republic of Congo: Findings from active safety surveillance,” for your renewed consideration as an original contribution in the Journal. As suggested by reviewers, we have addressed the comments from reviewers and included their insights in our manuscript.

Thank you for the opportunity to improve our manuscript by responding to the comments of the reviewers. Below we respond to specific comments and queries.

We look forward to your response.

Yours sincerely,

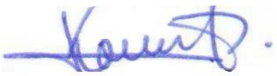

Kizito Kayumba, M.D., MSc

Africa Centers for Disease Control and Prevention

Addis Ababa, Ethiopia

Ph: +250 788 30 1834

KizitoK@africacdc.org

Journal Requirements:

If the reviewer comments include a recommendation to cite specific previously published works, please review and evaluate these publications to determine whether they are relevant and should be cited. There is no requirement to cite these works unless the editor has indicated otherwise.

Thank you for this insight, the reviewer comments do not include any recommendation to cite a specific previously published works

Please review your reference list to ensure that it is complete and correct. If you have cited papers that have been retracted, please include the rationale for doing so in the manuscript text, or remove these references and replace them with relevant current references. Any changes to the reference list should be mentioned in the rebuttal letter that accompanies your revised manuscript. If you need to cite a retracted article, indicate the article's retracted status in the References list and also include a citation and full reference for the retraction notice.

We thank the editor for drawing our attention to this. We have reviewed our reference list. We do not have any references that have been retracted. However, we made some changes to the reference list to address some typos errors and journal abbreviations:

On reference 2: We rectified a typos error in the title of the reference to become " *Vaccine-induced immune thrombotic thrombocytopenia (VITT) with massive pulmonary embolism and thrombocytopenia following a single dose of Janssen Ad26.COV2.S vaccination. Int J Infect Dis.* 2022;116:154-156." Instead of " ***A** Vaccine-induced massive pulmonary embolism and thrombocytopenia following a single dose of Janssen Ad26.COV2.S vaccination."*

On Reference 6: We replaced the abbreviation "WHO" by "World Health Organization" in the reference to be consistent with the journal abbreviations standard

[Note: HTML markup is below. Please do not edit.]

Reviewers' comments:

Reviewer's Responses to Questions

Comments to the Author

1. If the authors have adequately addressed your comments raised in a previous round of review and you feel that this manuscript is now acceptable for publication, you may indicate that here to bypass the “Comments to the Author” section, enter your conflict of interest statement in the “Confidential to Editor” section, and submit your "Accept" recommendation.

Reviewer #1: All comments have been addressed

Reviewer #2: (No Response)

Reviewer #3: All comments have been addressed

Reviewer #4: All comments have been addressed

Reviewer #5: (No Response)

---

2. Is the manuscript technically sound, and do the data support the conclusions?

The manuscript must describe a technically sound piece of scientific research with data that supports the conclusions. Experiments must have been conducted rigorously, with appropriate controls, replication, and sample sizes. The conclusions must be drawn appropriately based on the data presented.

Reviewer #1: Yes

Reviewer #2: Yes

Reviewer #3: Yes

Reviewer #4: Partly

Reviewer #5: Yes

---

3. Has the statistical analysis been performed appropriately and rigorously?

Reviewer #1: Yes

Reviewer #2: Yes

Reviewer #3: Yes

Reviewer #4: N/A

Reviewer #5: Yes

---

4. Have the authors made all data underlying the findings in their manuscript fully available?

The PLOS Data policy requires authors to make all data underlying the findings described in their manuscript fully available without restriction, with rare exception (please refer to the Data Availability Statement in the manuscript PDF file). The data should be provided as part of the manuscript or its supporting information, or deposited to a public repository. For example, in addition to summary statistics, the data points behind means, medians and variance measures should be available. If there are restrictions on publicly sharing data—e.g. participant privacy or use of data from a third party—those must be specified.

Reviewer #1: Yes

Reviewer #2: Yes

Reviewer #3: Yes

Reviewer #4: (No Response)

Reviewer #5: Yes

---

5. Is the manuscript presented in an intelligible fashion and written in standard English?

PLOS ONE does not copyedit accepted manuscripts, so the language in submitted articles must be clear, correct, and unambiguous. Any typographical or grammatical errors should be corrected at revision, so please note any specific errors here.

Reviewer #1: Yes

Reviewer #2: Yes

Reviewer #3: Yes

Reviewer #4: (No Response)

Reviewer #5: Yes

---

6. Review Comments to the Author

Please use the space provided to explain your answers to the questions above. You may also include additional comments for the author, including concerns about dual publication, research ethics, or publication ethics. (Please upload your review as an attachment if it exceeds 20,000 characters)

Reviewer #1: After detailed processing of the manuscript, taking into account the comments of the reviewers, an interesting study turned out with a logical construction of phrases and reasonable data obtained. All the questions were answered and reflected in the text of the article. One comment appeared regarding the paragraph "Findings from this study showed that the most common adverse event experienced among the majority after COVID-19 vaccination included fever (286 line) (458 cases), pain on injection site (429 cases), (287 line) headache (196 cases), stiffness (72 cases), and myalgia (55 cases). It is advisable for the authors to indicate the % of cases, since % is a more informative indicator for analysis.

We thank the reviewer for this valuable comment. We have expressed the incidence of these AEFIs in percentage

Reviewer #2: I reviewed the revised manuscript and I have no additional comments, all the comments have been addressed satisfactorily.

Reviewer #3: Very interesting article, nice to see this studies conducted since they are essential to ensure vaccine safety. All comments from previous reviewers as well as the editor's were addressed. My only comment would be that one of the reviewers suggested as a possible cause for the lower % of AEFIs reported compared to other studies may be the likelihood of previous exposure. While this is certainly a possible explanation, the authors have included it now as the most likely explanation in the discussion while it seems based on the Ghana study that premedication with paracetamol may be just as likely.

We thank the reviewer for this comment. As we have not assessed for premedication with paracetamol, we hypothesized that this may mask AEFI based on the Ghana study. However, previous exposure could have been impacted ton AEFI incidence as well, as the study participants had been exposed to the virus before vaccination. We kept the two hypotheses in our discussion and suggested for more evidence to explain these findings.

Reviewer #4: This may be of worth posting on the governmental institution's website or on their publications. However, I do not believe it is worthy of publication as an article in a peer-reviewed journal that is read by people all over the world.

This is because, although the risks of short-term, relatively common adverse reactions to the SARS-CoV-2 vaccine varies depending on the individual circumstances of the subjects, they do not much vary between countries around the world.
Therefore, if the risks of adverse reactions in the authors' country are significantly and greatly different from that of generally seen in other countries with careful discussion on the reasons of difference, it may be worthy of publication as an article in a peer-reviewed journal.
However, unless they cound such difference with thoughtful discussion, I do not believe it is worthy of publication as an article in a peer-reviewed journal.
I could neither find such difference, consequently nore careful discussions in this manuscript.

We thank the reviewer for their thoughtful critique. We respectfully disagree with the assessment and believe that our manuscript makes a critical contribution to the global literature, precisely by addressing the significant evidence gap in pharmacovigilance for underrepresented populations.

The reviewer suggests that AEFI risks do not vary greatly between countries. However, we are convinced that while the types of AEFIs may be similar, their incidence, severity, and risk factors cannot be assumed identical across different genetic and environmental backgrounds. Our study identifies a substantial and important difference: the overall AEFI incidence in our DRC cohort was 23.75%, which is markedly lower than the 65-80% reported in many Western studies (e.g., UK and US participant-reported data). This finding is not merely a minor variation but a significant discrepancy that warrants international attention and explanation.

In our discussion, we provide a careful and thoughtful analysis of potential reasons for this difference, directly addressing the reviewer's request. We hypothesize that a high rate of prior, undocumented SARS-CoV-2 infection in the DRC population may have led to a better-tolerated, non-primary immune response. We also cite regional practices, such as the widespread pre-medication with analgesics documented in the region, and methodological differences between our active surveillance and passive, app-based reporting systems that may over-represent AEFIs. This lower incidence has direct implications for public health communication and vaccine confidence in the region.

Beyond this key finding, the manuscript's value is multi-faceted. It provides the first robust, active safety surveillance data for a mass vaccination campaign in the DRC, filling a critical gap for a population underrepresented in initial clinical trials. It also offers locally relevant evidence on risk factors, demonstrating significantly lower odds of AEFIs in both the very

young and elderly within this specific demographic. The strongly decreased odds of AEFIs in those under 18 and over 60 provides reassuring local safety data for vulnerable age groups, supporting continued vaccination efforts for the elderly and paving the way for pediatric vaccination campaigns. Finally, it serves as a proof-of-concept for implementing advanced pharmacovigilance in a low-resource setting, a valuable methodological contribution for global health security.

We are confident that with revisions that more prominently highlight these comparative findings and their implications, the manuscript will be seen as a worthy contribution to the peer-reviewed literature, providing essential safety data for a global audience.

Reviewer #5: General impression
The manuscript addresses a highly relevant topic for public health, particularly considering the underrepresentation of African populations in previous COVID-19 vaccine trials. The active safety surveillance methodology strengthens the study and provides valuable evidence on adverse events following immunization in the Democratic Republic of Congo.

Strengths

Large and well-described sample (n=4766).

Active follow-up up to 28 days, improving data quality.

Inclusion of analyses by age group and vaccine type.

Discussion well contextualized with international literature.

Major comments

Clarity of English writing – Despite improvements, some sections (particularly Introduction and Discussion) remain difficult to read. A professional language revision is recommended.

We thank the reviewer for this thorough observation and recommendation. We have reviewed the introduction and discussion with a professional English language to make these sections easy to read.

Methodology – Participant selection – Clarify potential biases (e.g., exclusion of individuals without phone access), as this may affect representativeness.

We thank the reviewer and we concur with their observation that excluding individuals without phone access could constitute a selection bias, affecting representativeness. We have highlighted this in the manuscript, in the section of Limitations

Discussion – Pre-medication – The absence of paracetamol use assessment should be highlighted as a formal limitation.

We appreciate the suggestion of the reviewer to mention the absence of paracetamol use assessment as a formal limitation. We have highlighted this in the section of study limitations.

Rare adverse events – Specify whether unusual AEFIs (e.g., menstrual disturbances) were clinically verified or self-reported, as this impacts robustness of conclusions.

We thank the reviewer for emphasizing on how unusual adverse events were assessed. These were not clinically verified, which impacts robustness of conclusions. We have specified this in the limitation section as well.

Supplementary data – Provide full tables including less frequent AEFIs as supplementary material for transparency.

We appreciate the suggestion from reviewer to provide ***tables*** *including less frequent AEFIs. We have included the tables of less frequent AEFIs in S1 Table of Supplementary material.*

Minor comments

Adjust minor formatting inconsistencies in tables.

We thank the reviewer for this observation. We have thoroughly reviewed the tables and addressed formatting inconsistencies

Standardize terminology (use “AEFI” consistently).

We appreciate this comment and we have standardized the use of AEFI consistently

Reduce redundancy between Results and Discussion.

We thank the reviewer for this great insight. We managed to reduce redundancy between Results and Discussion by removing from discussion the information that was already provided in Results Section. We reshaped the Discussion Section to improve clarity and enable easy reading

Conclusion
This is a valuable study that would benefit from:

additional clarity in the text,

more detail on potential selection bias,

clear acknowledgment of limitations,

clarification of validation of rare AEFIs.

I recommend minor to moderate revision before publication.
