# Supplementary material for: Adverse events following immunization during COVID-19 mass vaccination campaigns in the Democratic Republic of Congo: Findings from active safety surveillance
Source: PLoS One. 2026 Jul 10;21(7):e0309628. doi: 10.1371/journal.pone.0309628 (PMC13353984; doi:10.1371/journal.pone.0309628)
Supplement: Attachment — Submitted filename: Responses_to_Reviewers_Comments_auresp_3.pdf [file pone.0309628.s013.pdf]

February 11, 2026

Dear Nuru Abdu

Enclosed is a revised version of our manuscript, titled: “Adverse events following immunization during COVID-19 mass vaccination campaigns in the Democratic Republic of Congo: Findings from active safety surveillance,” for your renewed consideration as an original contribution in the Journal. As suggested by reviewers, we have addressed the comments from reviewers and revised the manuscript appropriately.

Thank you for the opportunity to improve our manuscript by responding to the comments of the reviewers and revising our manuscript. Below we respond to specific comments and queries.

We look forward to your response.

Yours sincerely,

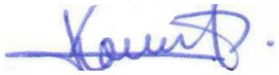

Kizito Kayumba, M.D., MSc

Ph: +250 788 30 1834

Email: kizitokayumba@gmail.com

Journal Requirements:

If the reviewer comments include a recommendation to cite specific previously published works, please review and evaluate these publications to determine whether they are relevant and should be cited. There is no requirement to cite these works unless the editor has indicated otherwise.

Thank you for this insight. The reviewer comments do not include any recommendation to cite a specific previously published works

Comments to the Author

1. If the authors have adequately addressed your comments raised in a previous round of review and you feel that this manuscript is now acceptable for publication, you may indicate that here to bypass the “Comments to the Author” section, enter your conflict of interest statement in the “Confidential to Editor” section, and submit your "Accept" recommendation.

Reviewer #1: All comments have been addressed

Reviewer #2: (No Response)

Reviewer #6: (No Response)

---

2. Is the manuscript technically sound, and do the data support the conclusions?

The manuscript must describe a technically sound piece of scientific research with data that supports the conclusions. Experiments must have been conducted rigorously, with appropriate controls, replication, and sample sizes. The conclusions must be drawn appropriately based on the data presented.

Reviewer #1: Yes

Reviewer #2: Yes

Reviewer #6: Yes

---

3. Has the statistical analysis been performed appropriately and rigorously?

Reviewer #1: Yes

Reviewer #2: Yes

Reviewer #6: Yes

---

4. Have the authors made all data underlying the findings in their manuscript fully available?

The PLOS Data policy requires authors to make all data underlying the findings described in their manuscript fully available without restriction, with rare exception (please refer to the Data Availability Statement in the manuscript PDF file). The data should be provided as part of the manuscript or its supporting information, or deposited to a public repository. For example, in addition to summary statistics, the data points behind means, medians and variance measures should be available. If there are restrictions on publicly sharing data—e.g. participant privacy or use of data from a third party—those must be specified.

Reviewer #1: Yes

Reviewer #2: Yes

Reviewer #6: Yes

---

5. Is the manuscript presented in an intelligible fashion and written in standard English?

PLOS ONE does not copyedit accepted manuscripts, so the language in submitted articles must be clear, correct, and unambiguous. Any typographical or grammatical errors should be corrected at revision, so please note any specific errors here.

Reviewer #1: Yes

Reviewer #2: Yes

Reviewer #6: Yes

---

6. Review Comments to the Author

Reviewer #1: All the existing comments were eliminated during the previous iterations of the review. There are no questions about the current edition of the manuscript

We thank the reviewer for confirming that all previous comments have been satisfactorily addressed.

Reviewer #2: I no additional comments for the authors. However, the comments raised by reviewer no. 4 are critical.

> *We thank the reviewer for this assessment. The comments raised by this Reviewer have been addressed in the revised manuscript, with clarifications and additions incorporated into the* **Discussion** *section.*

Reviewer #6: It is an interesting article; the methodology in which ad-hoc questionnaires on safety are conducted complements the data obtained through spontaneous reporting, favoring the acquisition of the complete safety profile of the drug when used under real conditions. The safety of vaccines used in the prevention of COVID is another relevant aspect. This methodology also allows us to understand how different variables affect the safety profile of these vaccines, such as age, the number of doses, and the time since administration.

> *We thank the reviewer for this positive and insightful evaluation of our work. We agree that active safety surveillance using structured questionnaires complements spontaneous reporting systems and provides a more comprehensive real-world safety profile.*

Methodology.

1) The low representativeness of patients under 18 years and over 65 years may be due to the type of recruitment through a telephone intervention, and this should be noted in the study's limitations.

> *We agree with the reviewer. As suggested, we have explicitly acknowledged this limitation in the* ***Limitations section****, noting that the telephone-based recruitment and follow-up approach may have introduced selection bias and reduced representativeness among younger and older age groups (<18 years and ≥60 years).*

2) Include the questionnaire in supplementary materials, is there any section for the patient's free response? what methodology was followed for its development?

> *We thank the reviewer for this important suggestion. The questionnaire has been included as* ***Supporting Information (S4 Table)****.The questionnaire was WHO-adapted with pre-defined AESIs and included an open field for additional participant-reported AEFIs (others to be specified), which were coded prior to analysis. We clarified this in the* ***Data collection and management*** *subsection*

Results

3) The sample is small in patients with the BNT162b2 vaccine and in age groups over 65 years and under 18 years; it would also be interesting to know the incidence of a second vaccine dose or heterologous vaccination. Has repeating the intervention at a later time to obtain this data been considered?

> *We thank the reviewer for this insight. We acknowledge this limitation and we clarified in the* ***Limitations section*** *that the low number of second-dose recipients and absence of heterologous vaccination limited our ability to conduct meaningful subgroup analyses. We have also added a statement noting that future or repeated active surveillance rounds will be required to assess second-dose and heterologous vaccination safety.*

4) The age range between 18 and 65 years is too wide; a more detailed analysis would allow seeing the reactivity in young patients compared to middle-aged patients.

> *We appreciate this suggestion. This point has been acknowledged in the* ***Limitations section****, noting that the broad age grouping (18–59 years) may have masked differences between younger and middle-aged adults and should be explored in future studies with larger samples.*

Discussion

5) Comment on the spontaneous reporting data in this country regarding these medications.
Include other studies with a similar methodology that allows knowing the safety profile of other medications.

> *We agree with the reviewer that active safety surveillance methodologies have been widely used to assess vaccine and medication safety beyond COVID-19 vaccines, particularly in post-marketing contexts. We expanded the Discussion and we have added a subsection entitled* ***"Active surveillance as a complement to spontaneous reporting"*** *into the* ***Discussion section*** *where we referenced additional studies using active or cohort-based surveillance approaches (including vaccine safety studies in Africa and other LMICs), highlighting methodological similarities and reinforcing the validity of our approach for evaluating real-world safety profiles.*

6) Discuss the advantages and disadvantages of a telephone intervention compared to an in-person one, and what possibilities there would be to carry it out in this context, for example, in community pharmacies.

We appreciate this insightful comment. In the ***Discussion*** *section, we have included a concise comparison of telephone-based versus in-person surveillance, outlining key advantages (feasibility, cost, scale) and limitations (selection bias, self-reporting, lack of clinical verification), and briefly discussed potential alternative community-based approaches within the DRC context.*

We thank all reviewers for their constructive feedback and careful evaluation of the manuscript. We believe that the revisions made have substantially improved the clarity, methodological transparency, and relevance of the study.
