# Supplementary material for: Adverse events following immunization during COVID-19 mass vaccination campaigns in the Democratic Republic of Congo: Findings from active safety surveillance
Source: PLoS One. 2026 Jul 10;21(7):e0309628. doi: 10.1371/journal.pone.0309628 (PMC13353984; doi:10.1371/journal.pone.0309628)
Supplement: Attachment — Submitted filename: Response to reviewers.doc [file pone.0309628.s011.doc]

May 14, 2025

Dear Nuru Abdu

Enclosed is a revised version of our manuscript, titled: “Adverse events following immunization during COVID-19 mass vaccination campaigns in the Democratic Republic of Congo: Findings from active safety surveillance,” for your renewed consideration as an original contribution in the Journal. As suggested, we have followed the manuscript preparation guidelines to align with Journal requirements.

Thank you for the opportunity to improve our manuscript by responding to the comments of the reviewers. Below we respond to specific comments and queries.

We look forward to your response.

Yours sincerely,

Kizito Kayumba, M.D., MSc

Africa Centers for Disease Control and Prevention

Addis Ababa, Ethiopia

Ph: +250 788 30 1834

KizitoK@africacdc.org

Journal Requirements:

When submitting your revision, we need you to address these additional requirements.

1. Please ensure that your manuscript meets PLOS ONE's style requirements, including those for file naming. The PLOS ONE style templates can be found at

<https://journals.plos.org/plosone/s/file?id=wjVg/PLOSOne_formatting_sample_main_body.pdf> and 
<https://journals.plos.org/plosone/s/file?id=ba62/PLOSOne_formatting_sample_title_authors_affiliations.pdf>

*As suggested by the editor, we have referred to the provided templates to format the manuscript*

2. We note that the grant information you provided in the ‘Funding Information’ and ‘Financial Disclosure’ sections do not match.

When you resubmit, please ensure that you provide the correct grant numbers for the awards you received for your study in the ‘Funding Information’ section.

*We appreciated the suggestion from the editor. However, there is no grant number for this funding and we highlighted this in the Cover letter. We revised the information provided in Funding information to match with information in Financial disclosure*

3. We note that you have indicated that there are restrictions to data sharing for this study. For studies involving human research participant data or other sensitive data, we encourage authors to share de-identified or anonymized data. However, when data cannot be publicly shared for ethical reasons, we allow authors to make their data sets available upon request. For information on unacceptable data access restrictions, please see [http://journals.plos.org/plosone/s/data-availability#loc-unacceptable-data-access-restrictions](http://journals.plos.org/plosone/s/data-availability#loc-unacceptable-data-access-restrictions).

Before we proceed with your manuscript, please address the following prompts:

a) If there are ethical or legal restrictions on sharing a de-identified data set, please explain them in detail (e.g., data contain potentially identifying or sensitive patient information, data are owned by a third-party organization, etc.) and who has imposed them (e.g., a Research Ethics Committee or Institutional Review Board, etc.). Please also provide contact information for a data access committee, ethics committee, or other institutional body to which data requests may be sent.

b) If there are no restrictions, please upload the minimal anonymized data set necessary to replicate your study findings to a stable, public repository and provide us with the relevant URLs, DOIs, or accession numbers. Please see <http://www.bmj.com/content/340/bmj.c181.long> for guidelines on how to de-identify and prepare clinical data for publication. For a list of recommended repositories, please see <https://journals.plos.org/plosone/s/recommended-repositories>. You also have the option of uploading the data as Supporting Information files, but we would recommend depositing data directly to a data repository if possible.

*We thank the editor for the guidance. We followed the guidelines to de-identify data and we have uploaded the minimal anonymized data set as Supporting Information files.*

Please update your Data Availability statement in the submission form accordingly.

*We appreciated the request from editor and we have updated the Data Availability statement*

4. For studies involving third-party data, we encourage authors to share any data specific to their analyses that they can legally distribute. PLOS recognizes, however, that authors may be using third-party data they do not have the rights to share. When third-party data cannot be publicly shared, authors must provide all information necessary for interested researchers to apply to gain access to the data. ([https://journals.plos.org/plosone/s/data-availability#loc-acceptable-data-access-restrictions](https://journals.plos.org/plosone/s/data-availability#loc-acceptable-data-access-restrictions))

For any third-party data that the authors cannot legally distribute, they should include the following information in their Data Availability Statement upon submission:
1) A description of the data set and the third-party source
2) If applicable, verification of permission to use the data set
3) Confirmation of whether the authors received any special privileges in accessing the data that other researchers would not have
4) All necessary contact information others would need to apply to gain access to the data

*As requested by editor, relevant information was provided.*

5. Please amend the manuscript submission data (via Edit Submission) to include author “Elizabeth Gonese”.

*We thank the editor for this observation. Elizabeth Gonese had started to contribute to this work. However, she later left the organization and we missed her contacts. So she could not continue to engage with the team and did not contribute equally to the work. Therefore we could not include her among the authors*

**Additional Editor Comments:**

Your manuscript does not follow the PLOS ONE manuscript preparation guidelines. I suggest the authors follow the author manuscript preparation guidelines using the following link (<https://journals.plos.org/plosone/s/submission-guidelines>)

*We thank the editor for the guidance and the many comments which we have now addressed throughout the manuscript. The paper has been significantly strengthened by incorporation of the changes based on editor’s guidance.*

5. Review Comments to the Author

Please use the space provided to explain your answers to the questions above. You may also include additional comments for the author, including concerns about dual publication, research ethics, or publication ethics. (Please upload your review as an attachment if it exceeds 20,000 characters)

Reviewer #1: The research design was constructed competently, the analysis of the obtained results and the description of the material are logical. However, there are some comments:

1) Line 126-128: Please explain why you considered the age of dichotomization into groups to be 36 years old?

*We restructured the age groups to focus on those documented by other authors in the literature to have impact on AEFI occurrence. The new categories of age considered are the age groups of <18 years, 18-59 years, ≥60 years which correspond to age groups with different type of immune system response. The previous dichotomization of age was based on median age of our participants, which is 36 years.*

2) Line 166-167: Why the timing of the occurrence of reactions and its duration are not indicated? What is about description of first aid measures? This will help to reveal the severity of the course of such reactions and pay more attention to the warnings in future. In my opinion, it is important to add.

*As suggested by the reviewer, we added information about time of occurance of reactions in the Results, Incidence and forms of AEFIs reported Section. We also added a description of measures followed for any SAE detected. This was placed in Methods, Data collection and management Section*

3) Line 180-183: There is a wide range in age from 12 years to 60+. In this regard, why was the tolerance of vaccination in people aged 60+, teenagers 12-17 and children less than 12 years old (as extreme in age with absolutely different type of immune system response) not taken into account differently but in two big and heterogeneous groups? The graphs with the age 60+ and <18 should be added and counted.

*We appreciated the reviewer for the relevant comments and suggestions. We structured the age groups to consider the extreme age (60+ and <18 ) and we repeated the analysis. The new age groups categories are <18 years, 18-59 years and ≥60 years. We included new findings throughout the manuscript.*

4) Line 229: The final picture or table is missing - only for reliable and statistically significant differences in the development of AEFI with inclusion of fever, vomiting, headache, muscle pain, joint pain, body pain, pain at the injection site, diarrhea and chills depending on the vaccine preparation.

*We appreciated the comment by the reviewer and we have added the table showing different forms of AEFIs depending on the vaccine preparation. We have compiled the content of different tables and reduced the number of tables by presenting the most relevant tables. We remained with 4 tables in the manuscript and other tables were presented in supporting information.*

5) Line 248-249: It would be nice to add possible reason for this. It is likely that all those vaccinated were previously infected with SARS-CoV-2 (period of inclusion - December 2023), and in this case a repeated encounter with the vaccine antigen is not primary, therefore tolerance is better.

*We thank the reviewer for the great suggestion, we have included this additional reason to the manuscript in Discussion Section*

6) Line 301-304: This is new, not previously described in any study adverse effect that needs to be reflected in the list of AEFI in the future recommendations for clinical trials of vaccines of various production technologies.

*As noted by the reviewer, menstrual disorders and unexpected vaginal bleeding is a new adverse effect and we have included it in the Discussion. We recommended that it should be carefully monitored in future surveys.*

7) Line 317: Where are from this limitations - 5−90 years? Previously in the text it was written only about 60+ and <12 years old.

*We appreciate observation from reviewer. These limitations of 5−90 years are minimum age and maximum age of participants to the study. We have included this information in Result section to align with what is found in Conclusion. The group of 60+ and <12 years old are age group categories previously used in descriptive analysis.*

8) Line 318: “Transient” - there is nothing in the text about the duration of reaction persistence (see point 2)

*As suggested by the reviewer, we have included information about the duration of reaction persistence in Results Section. We also added the figure 2 highlighting the timing of AEFIs occurrence and persistence; and the figure 3 for the different forms of AEFIs (desaggregated) and duration of their persistence*

Taking into account the above comments, it is also necessary to change and improve the abstract with new data.

*We thank the reviewer for the many comments and suggestions which we have now addressed throughout the manuscript. The paper has been significantly strengthened by incorporation of these changes. We have improved the abstract accordingly*

Reviewer #2: General Feedback:
• Please revise the manuscript to enhance the English throughout, including the abstract.

*We appreciated the comment by the reviewer, we have refined the English and made edits throughout the manuscript including the abstract.*

• Minimize the use of "we" throughout the manuscript.

*As suggested by the reviewer, we reviewed the manuscript to minimize the use of “we”.*

• Replace manufacturer names with vaccine brand names (if applicable).

*We appreciated the comment and we replaced the manufacturer name by the brand names commonly used.*

Specific Comments:
• Africa CDC's Role: Please clarify the specific role of the Africa CDC in the analysis. Justify their involvement and explain why a statistician or independent researchers weren't used instead. Remove "Africa CDC" from the keywords.

*The Africa CDC’s role in the analysis is that a data analyst at Africa CDC supported analysis. We have revised the section accordingly. As suggested by the reviewer, Africa CDC has been removed from key words.*

• Supporting Evidence (Line 55-56): Strengthen the claim by providing additional robust references.

*As suggested by the reviewer, we have revised literature. We have included additional reference.*

• Primary References (Lines 61-64): Please use primary references instead of cross-referencing.

*As suggested by the reviewer, we have reviewed the references and used the primary reference*

• Reference 5: Provide the complete reference details in the reference section.

*We appreciated the comment from the reviewer and we have provided the complete reference details in reference section.*

• Participant Selection and Sampling: Describe the participant selection process in detail, including the sampling technique used and how the sample size was calculated.

*We appreciated the comment by the reviewer and have renamed the sub-section “Study participants” to “Participant Selection and Sampling” in the Methods and added a more precise and detailed description of the participant selection process. Description of* *the sampling technique and sample size calculation was provided in a different section called “Sampling technique and sample size”*

• Sampling Technique and Sample Size: Clearly describe the sampling technique and justify the chosen sample size.

*We have added Sampling Technique and Sample Size sub-section where we described in details the sampling technique used and the sample size calculation*

• Introduction and Setting (Lines 85-98): Move the content from lines 85-98 to the introduction and provide a more detailed description of the study setting.

*We have removed this content as suggested by the reviewer and we added more information in the Introduction and Setting section to clarify the study setting*

• Clearly define the outcome variables using a separate sub-section.

*We appreciated the suggestion from the reviewer and we have revised the data analysis section to better clarify outcome variables. We have placed this content in a separate sub-section of outcome variables*

• Data Collection Tool: Please provide the data collection tool as a supplementary file.

*Data collection tool was provided as supplementary file in Supporting information Section*

• Reference Sequencing: The reference sequencing appears incorrect. There's a jump from reference 9 (line 98) to reference 14 (line 126). Please verify and correct.

*As noted by the reviewer, the jump in references has been verified and corrected*

• Dichotomization of Categorical Variables: Provide references that discuss the dichotomization of multiple categorical variables in detail, including the specific statistical methods used instead of the used reference.

*As noted by the reviewer, we provided references related to dichotomization of categorical variables in detail in Methods and in discussion section.*

• Multivariable vs. Multivariate (Line 132): Use "multivariable" instead of "multivariate" consistently throughout the manuscript, as your model involves one dependent variable and multiple independent variables.

*As suggested by the reviewer, we consistently used "multivariable" throughout the manuscript*

• Sex vs. Gender: Maintain consistent use of "sex" instead of "gender," given that only clinically relevant variables were collected.

*As suggested by the reviewer, we consistently used "sex" instead of "gender," throughout the manuscript*

• Missing Participant (Vaccine Type and Age groups): Explain why one participant is missing in the vaccine type variable, and how this relates to the age group stratification.

*As noted by the reviewer, we revised the table of participants and vaccine characteristics to rectify any inconsistency. Under Vaccine Type, one participant seemed to be missing because of typing error.*

• Bivariate Analysis: Explain why bivariate analysis was not conducted before the multivariable analysis.

*Bivariate analysis was conducted but we only presented results from the multivariable logistic regression analysis as we aimed to control for confounding. We controlled for confounding through the use of multivariable analysis. This is described in the Methods, Data Analysis sub-section.*

• AEFI Incidence (Lines 180-183): Move the content from lines 180-183 to the incidence of AEFI sub-section of the findings.

*As noted by the reviewer, we have moved this content to the incidence of AEFI sub-section of the results.*

• Effect Size and Logistic Regression Assumptions: Given the smaller sample size for the Pfizer group compared to the Johnson & Johnson vaccine group, please supplement the analysis with effect size calculations. Alternatively, justify the use of logistic regression by confirming that the data met the necessary assumptions for accurate results.

*Though there is a smaller sample size for Pfizer group compared to the Johnson & Johnson vaccine group, there is a minimum number of necessary samples to meet the desired statistical constraints. We controlled for basic assumptions that must be met for logistic regression that include independence of errors, linearity in the logit for continuous variables, absence of multicollinearity, and lack of strongly influential outliers.* *Several tests were performed to test the fitness of data in our logistic regression model. The Chi-square test of our model is statistically significant (p <0.001), which means that the overall model fits. Therefore odds ratios were used as measures of effect size.* *The Hosmer-Lemeshow statistic indicates a poor fit if the significance value is less than 0.05 According to the goodness-of-fit test for our logistic regression model, the model adequately fits the data (p> 0.45). Multicollinearity was assessed by VIF=1 suggesting no multicollinearity.*

• Table 6 Omitted Variable: Add a footnote to Table 6 explaining why the specific variable was omitted from the analysis.

*As suggested by the reviewer, we have added a footnote to Table 6 and we explained why the variable was omitted from the analysis.*
