# Supplementary material for: Adverse events following immunization during COVID-19 mass vaccination campaigns in the Democratic Republic of Congo: Findings from active safety surveillance
Source: PLoS One. 2026 Jul 10;21(7):e0309628. doi: 10.1371/journal.pone.0309628 (PMC13353984; doi:10.1371/journal.pone.0309628)
Supplement: Attachment — Submitted filename: Review result.docx [file pone.0309628.s010.docx]

Review result: reject

Main reasons

This may be of worth posting on the governmental institution's website or on their publications. However, I do not believe it is worthy of publication as an article in a peer-reviewed journal that is read by people all over the world.

This is because, although the risks of short-term, relatively common adverse reactions to the SARS-CoV-2 vaccine varies depending on the individual circumstances of the subjects, they do not much vary between countries around the world.

Therefore, if the risks of adverse reactions in the authors’ country are significantly and greatly different from that of generally seen in other countries with careful discussion on the reasons of difference, it may be worthy of publication as an article in a peer-reviewed journal.

However, unless they cound such difference with thoughtful discussion, I do not believe it is worthy of publication as an article in a peer-reviewed journal.

I could neither find such difference, consequently nore careful discussions in this manuscript.
