# Supplementary material for: Adverse events following immunization during COVID-19 mass vaccination campaigns in the Democratic Republic of Congo: Findings from active safety surveillance
Source: PLoS One. 2026 Jul 10;21(7):e0309628. doi: 10.1371/journal.pone.0309628 (PMC13353984; doi:10.1371/journal.pone.0309628)
Supplement: Peer review history

# Peer review: Decision Letter 0

**DOI:** 10.1371/journal.pone.0309628.r001

**Authors:** Nuru Abdu

3 Apr 2025

PONE-D-24-31631

Adverse events following immunization during COVID-19 mass vaccination campaigns in the Democratic Republic of Congo: Findings from active safety surveillance

PLOS ONE

Dear Dr. Kayumba,

Thank you for submitting your manuscript to PLOS ONE. After careful consideration, we feel that it has merit but does not fully meet PLOS ONE’s publication criteria as it currently stands. Therefore, we invite you to submit a revised version of the manuscript that addresses the points raised during the review process.

Please submit your revised manuscript by May 15 2025 11:59PM. If you will need more time than this to complete your revisions, please reply to this message or contact the journal office at plosone@plos.org. When you're ready to submit your revision, log on to https://www.editorialmanager.com/pone/ and select the 'Submissions Needing Revision' folder to locate your manuscript file.

Please include the following items when submitting your revised manuscript:

- A rebuttal letter that responds to each point raised by the academic editor and reviewer(s). You should upload this letter as a separate file labeled 'Response to Reviewers'.
- A marked-up copy of your manuscript that highlights changes made to the original version. You should upload this as a separate file labeled 'Revised Manuscript with Track Changes'.
- An unmarked version of your revised paper without tracked changes. You should upload this as a separate file labeled 'Manuscript'.

If you would like to make changes to your financial disclosure, please include your updated statement in your cover letter. Guidelines for resubmitting your figure files are available below the reviewer comments at the end of this letter.

If applicable, we recommend that you deposit your laboratory protocols in protocols.io to enhance the reproducibility of your results. Protocols.io assigns your protocol its own identifier (DOI) so that it can be cited independently in the future. For instructions see: https://journals.plos.org/plosone/s/submission-guidelines#loc-laboratory-protocols. Additionally, PLOS ONE offers an option for publishing peer-reviewed Lab Protocol articles, which describe protocols hosted on protocols.io. Read more information on sharing protocols at https://plos.org/protocols?utm_medium=editorial-email&utm_source=authorletters&utm_campaign=protocols.

We look forward to receiving your revised manuscript.

Kind regards,

Nuru Abdu, BPharm

Academic Editor

PLOS ONE

Journal Requirements:

When submitting your revision, we need you to address these additional requirements.

1. Please ensure that your manuscript meets PLOS ONE's style requirements, including those for file naming. The PLOS ONE style templates can be found at

https://journals.plos.org/plosone/s/file?id=wjVg/PLOSOne_formatting_sample_main_body.pdf and

https://journals.plos.org/plosone/s/file?id=ba62/PLOSOne_formatting_sample_title_authors_affiliations.pdf

2. We note that the grant information you provided in the ‘Funding Information’ and ‘Financial Disclosure’ sections do not match.

When you resubmit, please ensure that you provide the correct grant numbers for the awards you received for your study in the ‘Funding Information’ section.

3. We note that you have indicated that there are restrictions to data sharing for this study. For studies involving human research participant data or other sensitive data, we encourage authors to share de-identified or anonymized data. However, when data cannot be publicly shared for ethical reasons, we allow authors to make their data sets available upon request. For information on unacceptable data access restrictions, please see http://journals.plos.org/plosone/s/data-availability#loc-unacceptable-data-access-restrictions.

Before we proceed with your manuscript, please address the following prompts:

a) If there are ethical or legal restrictions on sharing a de-identified data set, please explain them in detail (e.g., data contain potentially identifying or sensitive patient information, data are owned by a third-party organization, etc.) and who has imposed them (e.g., a Research Ethics Committee or Institutional Review Board, etc.). Please also provide contact information for a data access committee, ethics committee, or other institutional body to which data requests may be sent.

b) If there are no restrictions, please upload the minimal anonymized data set necessary to replicate your study findings to a stable, public repository and provide us with the relevant URLs, DOIs, or accession numbers. Please see http://www.bmj.com/content/340/bmj.c181.long for guidelines on how to de-identify and prepare clinical data for publication. For a list of recommended repositories, please see https://journals.plos.org/plosone/s/recommended-repositories. You also have the option of uploading the data as Supporting Information files, but we would recommend depositing data directly to a data repository if possible.

Please update your Data Availability statement in the submission form accordingly.

4. For studies involving third-party data, we encourage authors to share any data specific to their analyses that they can legally distribute. PLOS recognizes, however, that authors may be using third-party data they do not have the rights to share. When third-party data cannot be publicly shared, authors must provide all information necessary for interested researchers to apply to gain access to the data. (https://journals.plos.org/plosone/s/data-availability#loc-acceptable-data-access-restrictions)

For any third-party data that the authors cannot legally distribute, they should include the following information in their Data Availability Statement upon submission:

1) A description of the data set and the third-party source

2) If applicable, verification of permission to use the data set

3) Confirmation of whether the authors received any special privileges in accessing the data that other researchers would not have

4) All necessary contact information others would need to apply to gain access to the data

5. Please amend the manuscript submission data (via Edit Submission) to include author “Elizabeth Gonese”.

**Additional Editor Comments:**

Your manuscript does not follow the PLOS ONE manuscript preparation guidelines. I suggest the authors follow the author manuscript preparation guidelines using the following link (https://journals.plos.org/plosone/s/submission-guidelines)

[Note: HTML markup is below. Please do not edit.]

Reviewers' comments:

Reviewer's Responses to Questions

**Comments to the Author**

1. Is the manuscript technically sound, and do the data support the conclusions?

The manuscript must describe a technically sound piece of scientific research with data that supports the conclusions. Experiments must have been conducted rigorously, with appropriate controls, replication, and sample sizes. The conclusions must be drawn appropriately based on the data presented.

Reviewer #1: Yes

Reviewer #2: Yes

**********

2. Has the statistical analysis been performed appropriately and rigorously?

Reviewer #1: Yes

Reviewer #2: No

**********

3. Have the authors made all data underlying the findings in their manuscript fully available?

The PLOS Data policy requires authors to make all data underlying the findings described in their manuscript fully available without restriction, with rare exception (please refer to the Data Availability Statement in the manuscript PDF file). The data should be provided as part of the manuscript or its supporting information, or deposited to a public repository. For example, in addition to summary statistics, the data points behind means, medians and variance measures should be available. If there are restrictions on publicly sharing data—e.g. participant privacy or use of data from a third party—those must be specified.

Reviewer #1: Yes

Reviewer #2: No

**********

4. Is the manuscript presented in an intelligible fashion and written in standard English?

PLOS ONE does not copyedit accepted manuscripts, so the language in submitted articles must be clear, correct, and unambiguous. Any typographical or grammatical errors should be corrected at revision, so please note any specific errors here.

Reviewer #1: Yes

Reviewer #2: No

**********

5. Review Comments to the Author

Please use the space provided to explain your answers to the questions above. You may also include additional comments for the author, including concerns about dual publication, research ethics, or publication ethics. (Please upload your review as an attachment if it exceeds 20,000 characters)

Reviewer #1: The research design was constructed competently, the analysis of the obtained results and the description of the material are logical. However, there are some comments:

1) Line 126-128: Please explain why you considered the age of dichotomization into groups to be 36 years old?

2) Line 166-167: Why the timing of the occurrence of reactions and its duration are not indicated? What is about description of first aid measures? This will help to reveal the severity of the course of such reactions and pay more attention to the warnings in future. In my opinion, it is important to add.

3) Line 180-183: There is a wide range in age from 12 years to 60+. In this regard, why was the tolerance of vaccination in people aged 60+, teenagers 12-17 and children less than 12 years old (as extreme in age with absolutely different type of immune system response) not taken into account differently but in two big and heterogeneous groups? The graphs with the age 60+ and <18 should be added and counted.

4) Line 229: The final picture or table is missing - only for reliable and statistically significant differences in the development of AEFI with inclusion of fever, vomiting, headache, muscle pain, joint pain, body pain, pain at the injection site, diarrhea and chills depending on the vaccine preparation.

5) Line 248-249: It would be nice to add possible reason for this. It is likely that all those vaccinated were previously infected with SARS-CoV-2 (period of inclusion - December 2023), and in this case a repeated encounter with the vaccine antigen is not primary, therefore tolerance is better.

6) Line 301-304: This is new, not previously described in any study adverse effect that needs to be reflected in the list of AEFI in the future recommendations for clinical trials of vaccines of various production technologies.

7) Line 317: Where are from this limitations - 5−90 years? Previously in the text it was written only about 60+ and <12 years old.

8) Line 318: “Transient” - there is nothing in the text about the duration of reaction persistence (see point 2)

Taking into account the above comments, it is also necessary to change and improve the abstract with new data.

Reviewer #2: General Feedback:

• Please revise the manuscript to enhance the English throughout, including the abstract.

• Minimize the use of "we" throughout the manuscript.

• Replace manufacturer names with vaccine brand names (if applicable).

Specific Comments:

• Africa CDC's Role: Please clarify the specific role of the Africa CDC in the analysis. Justify their involvement and explain why a statistician or independent researchers weren't used instead. Remove "Africa CDC" from the keywords.

• Supporting Evidence (Line 55-56): Strengthen the claim by providing additional robust references.

• Primary References (Lines 61-64): Please use primary references instead of cross-referencing.

• Reference 5: Provide the complete reference details in the reference section.

• Participant Selection and Sampling: Describe the participant selection process in detail, including the sampling technique used and how the sample size was calculated.

• Sampling Technique and Sample Size: Clearly describe the sampling technique and justify the chosen sample size.

• Introduction and Setting (Lines 85-98): Move the content from lines 85-98 to the introduction and provide a more detailed description of the study setting.

• Clearly define the outcome variables using a separate sub-section.

• Data Collection Tool: Please provide the data collection tool as a supplementary file.

• Reference Sequencing: The reference sequencing appears incorrect. There's a jump from reference 9 (line 98) to reference 14 (line 126). Please verify and correct.

• Dichotomization of Categorical Variables: Provide references that discuss the dichotomization of multiple categorical variables in detail, including the specific statistical methods used instead of the used reference.

• Multivariable vs. Multivariate (Line 132): Use "multivariable" instead of "multivariate" consistently throughout the manuscript, as your model involves one dependent variable and multiple independent variables.

• Sex vs. Gender: Maintain consistent use of "sex" instead of "gender," given that only clinically relevant variables were collected.

• Missing Participant (Vaccine Type and Age groups): Explain why one participant is missing in the vaccine type variable, and how this relates to the age group stratification.

• Bivariate Analysis: Explain why bivariate analysis was not conducted before the multivariable analysis.

• AEFI Incidence (Lines 180-183): Move the content from lines 180-183 to the incidence of AEFI sub-section of the findings.

• Effect Size and Logistic Regression Assumptions: Given the smaller sample size for the Pfizer group compared to the Johnson & Johnson vaccine group, please supplement the analysis with effect size calculations. Alternatively, justify the use of logistic regression by confirming that the data met the necessary assumptions for accurate results.

• Table 6 Omitted Variable: Add a footnote to Table 6 explaining why the specific variable was omitted from the analysis.

**********

6. PLOS authors have the option to publish the peer review history of their article (what does this mean?). If published, this will include your full peer review and any attached files.

If you choose “no”, your identity will remain anonymous but your review may still be made public.

**Do you want your identity to be public for this peer review?**  For information about this choice, including consent withdrawal, please see our Privacy Policy.

Reviewer #1: **Yes:** Kostinov Mikhail Petrovich Honored Scientist of the Russian Federation, Corresponding Member of the Russian Academy of Sciences, Professor, Head of the Department of Epidemiology and Modern Vaccination Technologies of the Professional Education Institute of the I.M. First Moscow State Medical University (Sechenov University);Head of the Laboratory of Vaccine Prophylaxis and Immunotherapy of Allergic Diseases of the I.I. Mechnikov Research Institute of Vaccines and Serums

Reviewer #2: No

**********

[NOTE: If reviewer comments were submitted as an attachment file, they will be attached to this email and accessible via the submission site. Please log into your account, locate the manuscript record, and check for the action link "View Attachments". If this link does not appear, there are no attachment files.]

While revising your submission, please upload your figure files to the Preflight Analysis and Conversion Engine (PACE) digital diagnostic tool, https://pacev2.apexcovantage.com/. PACE helps ensure that figures meet PLOS requirements. To use PACE, you must first register as a user. Registration is free. Then, login and navigate to the UPLOAD tab, where you will find detailed instructions on how to use the tool. If you encounter any issues or have any questions when using PACE, please email PLOS at figures@plos.org. Please note that Supporting Information files do not need this step.

# Author comment: Author response to Decision Letter 1

**DOI:** 10.1371/journal.pone.0309628.r002

27 May 2025

Journal Requirements:

When submitting your revision, we need you to address these additional requirements.

1. Please ensure that your manuscript meets PLOS ONE's style requirements, including those for file naming. The PLOS ONE style templates can be found at

https://journals.plos.org/plosone/s/file?id=wjVg/PLOSOne_formatting_sample_main_body.pdf and

https://journals.plos.org/plosone/s/file?id=ba62/PLOSOne_formatting_sample_title_authors_affiliations.pdf

As suggested by the editor, we have referred to the provided templates to format the manuscript

2. We note that the grant information you provided in the ‘Funding Information’ and ‘Financial Disclosure’ sections do not match.

When you resubmit, please ensure that you provide the correct grant numbers for the awards you received for your study in the ‘Funding Information’ section.

We appreciated the suggestion from the editor. However, there is no grant number for this funding and we highlighted this in the Cover letter. We revised the information provided in Funding information to match with information in Financial disclosure

3. We note that you have indicated that there are restrictions to data sharing for this study. For studies involving human research participant data or other sensitive data, we encourage authors to share de-identified or anonymized data. However, when data cannot be publicly shared for ethical reasons, we allow authors to make their data sets available upon request. For information on unacceptable data access restrictions, please see http://journals.plos.org/plosone/s/data-availability#loc-unacceptable-data-access-restrictions.

Before we proceed with your manuscript, please address the following prompts:

a) If there are ethical or legal restrictions on sharing a de-identified data set, please explain them in detail (e.g., data contain potentially identifying or sensitive patient information, data are owned by a third-party organization, etc.) and who has imposed them (e.g., a Research Ethics Committee or Institutional Review Board, etc.). Please also provide contact information for a data access committee, ethics committee, or other institutional body to which data requests may be sent.

b) If there are no restrictions, please upload the minimal anonymized data set necessary to replicate your study findings to a stable, public repository and provide us with the relevant URLs, DOIs, or accession numbers. Please see http://www.bmj.com/content/340/bmj.c181.long for guidelines on how to de-identify and prepare clinical data for publication. For a list of recommended repositories, please see https://journals.plos.org/plosone/s/recommended-repositories. You also have the option of uploading the data as Supporting Information files, but we would recommend depositing data directly to a data repository if possible.

We thank the editor for the guidance. We followed the guidelines to de-identify data and we have uploaded the minimal anonymized data set as Supporting Information files.

Please update your Data Availability statement in the submission form accordingly.

We appreciated the request from editor and we have updated the Data Availability statement

4. For studies involving third-party data, we encourage authors to share any data specific to their analyses that they can legally distribute. PLOS recognizes, however, that authors may be using third-party data they do not have the rights to share. When third-party data cannot be publicly shared, authors must provide all information necessary for interested researchers to apply to gain access to the data. (https://journals.plos.org/plosone/s/data-availability#loc-acceptable-data-access-restrictions)

For any third-party data that the authors cannot legally distribute, they should include the following information in their Data Availability Statement upon submission:

1) A description of the data set and the third-party source

2) If applicable, verification of permission to use the data set

3) Confirmation of whether the authors received any special privileges in accessing the data that other researchers would not have

4) All necessary contact information others would need to apply to gain access to the data

As requested by editor, relevant information was provided.

5. Please amend the manuscript submission data (via Edit Submission) to include author “Elizabeth Gonese”.

We thank the editor for this observation. Elizabeth Gonese had started to contribute to this work. However, she later left the organization and we missed her contacts. So she could not continue to engage with the team and did not contribute equally to the work. Therefore we could not include her among the authors

Additional Editor Comments:

Your manuscript does not follow the PLOS ONE manuscript preparation guidelines. I suggest the authors follow the author manuscript preparation guidelines using the following link (https://journals.plos.org/plosone/s/submission-guidelines)

We thank the editor for the guidance and the many comments which we have now addressed throughout the manuscript. The paper has been significantly strengthened by incorporation of the changes based on editor’s guidance.

5. Review Comments to the Author

Please use the space provided to explain your answers to the questions above. You may also include additional comments for the author, including concerns about dual publication, research ethics, or publication ethics. (Please upload your review as an attachment if it exceeds 20,000 characters)

Reviewer #1: The research design was constructed competently, the analysis of the obtained results and the description of the material are logical. However, there are some comments:

1) Line 126-128: Please explain why you considered the age of dichotomization into groups to be 36 years old?

We restructured the age groups to focus on those documented by other authors in the literature to have impact on AEFI occurrence. The new categories of age considered are the age groups of <18 years, 18-59 years, ≥60 years which correspond to age groups with different type of immune system response. The previous dichotomization of age was based on median age of our participants, which is 36 years.

2) Line 166-167: Why the timing of the occurrence of reactions and its duration are not indicated? What is about description of first aid measures? This will help to reveal the severity of the course of such reactions and pay more attention to the warnings in future. In my opinion, it is important to add.

As suggested by the reviewer, we added information about time of occurance of reactions in the Results, Incidence and forms of AEFIs reported Section. We also added a description of measures followed for any SAE detected. This was placed in Methods, Data collection and management Section

3) Line 180-183: There is a wide range in age from 12 years to 60+. In this regard, why was the tolerance of vaccination in people aged 60+, teenagers 12-17 and children less than 12 years old (as extreme in age with absolutely different type of immune system response) not taken into account differently but in two big and heterogeneous groups? The graphs with the age 60+ and <18 should be added and counted.

We appreciated the reviewer for the relevant comments and suggestions. We structured the age groups to consider the extreme age (60+ and <18 ) and we repeated the analysis. The new age groups categories are <18 years, 18-59 years and ≥60 years. We included new findings throughout the manuscript.

4) Line 229: The final picture or table is missing - only for reliable and statistically significant differences in the development of AEFI with inclusion of fever, vomiting, headache, muscle pain, joint pain, body pain, pain at the injection site, diarrhea and chills depending on the vaccine preparation.

We appreciated the comment by the reviewer and we have added the table showing different forms of AEFIs depending on the vaccine preparation. We have compiled the content of different tables and reduced the number of tables by presenting the most relevant tables. We remained with 4 tables in the manuscript and other tables were presented in supporting information.

5) Line 248-249: It would be nice to add possible reason for this. It is likely that all those vaccinated were previously infected with SARS-CoV-2 (period of inclusion - December 2023), and in this case a repeated encounter with the vaccine antigen is not primary, therefore tolerance is better.

We thank the reviewer for the great suggestion, we have included this additional reason to the manuscript in Discussion Section

6) Line 301-304: This is new, not previously described in any study adverse effect that needs to be reflected in the list of AEFI in the future recommendations for clinical trials of vaccines of various production technologies.

As noted by the reviewer, menstrual disorders and unexpected vaginal bleeding is a new adverse effect and we have included it in the Discussion. We recommended that it should be carefully monitored in future surveys.

7) Line 317: Where are from this limitations - 5−90 years? Previously in the text it was written only about 60+ and <12 years old.

We appreciate observation from reviewer. These limitations of 5−90 years are minimum age and maximum age of participants to the study. We have included this information in Result section to align with what is found in Conclusion. The group of 60+ and <12 years old are age group categories previously used in descriptive analysis.

8) Line 318: “Transient” - there is nothing in the text about the duration of reaction persistence (see point 2)

As suggested by the reviewer, we have included information about the duration of reaction persistence in Results Section. We also added the figure 2 highlighting the timing of AEFIs occurrence and persistence; and the figure 3 for the different forms of AEFIs (desaggregated) and duration of their persistence

Taking into account the above comments, it is also necessary to change and improve the abstract with new data.

We thank the reviewer for the many comments and suggestions which we have now addressed throughout the manuscript. The paper has been significantly strengthened by incorporation of these changes. We have improved the abstract accordingly

Reviewer #2: General Feedback:

• Please revise the manuscript to enhance the English throughout, including the abstract.

We appreciated the comment by the reviewer, we have refined the English and made edits throughout the manuscript including the abstract.

• Minimize the use of "we" throughout the manuscript.

As suggested by the reviewer, we reviewed the manuscript to minimize the use of “we”.

• Replace manufacturer names with vaccine brand names (if applicable).

We appreciated the comment and we replaced the manufacturer name by the brand names commonly used.

Specific Comments:

• Africa CDC's Role: Please clarify the specific role of the Africa CDC in the analysis. Justify their involvement and explain why a statistician or independent researchers weren't used instead. Remove "Africa CDC" from the keywords.

The Africa CDC’s role in the analysis is that a data analyst at Africa CDC supported analysis. We have revised the section accordingly. As suggested by the reviewer, Africa CDC has been removed from key words.

• Supporting Evidence (Line 55-56): Strengthen the claim by providing additional robust references.

As suggested by the reviewer, we have revised literature. We have included additional reference.

• Primary References (Lines 61-64): Please use primary references instead of cross-referencing.

As suggested by the reviewer, we have reviewed the references and used the primary reference

• Reference 5: Provide the complete reference details in the reference section.

We appreciated the comment from the reviewer and we have provided the complete reference details in reference section.

• Participant Selection and Sampling: Describe the participant selection process in detail, including the sampling technique used and how the sample size was calculated.

We appreciated the comment by the reviewer and have renamed the sub-section “Study participants” to “Participant Selection and Sampling” in the Methods and added a more precise and detailed description of the participant selection process. Description of the sampling technique and sample size calculation was provided in a different section called “Sampling technique and sample size”

• Sampling Technique and Sample Size: Clearly describe the sampling technique and justify the chosen sample size.

We have added Sampling Technique and Sample Size sub-section where we described in details the sampling technique used and the sample size calculation

• Introduction and Setting (Lines 85-98): Move the content from lines 85-98 to the introduction and provide a more detailed description of the study setting.

We have removed this content as suggested by the reviewer and we added more information in the Introduction and Setting section to clarify the study setting

• Clearly define the outcome variables using a separate sub-section.

We appreciated the suggestion from the reviewer and we have revised the data analysis section to better clarify outcome variables. We have placed this content in a separate sub-section of outcome variables

• Data Collection Tool: Please provide the data collection tool as a supplementary file.

Data collection tool was provided as supplementary file in Supporting information Section

• Reference Sequencing: The reference sequencing appears incorrect. There's a jump from reference 9 (line 98) to reference 14 (line 126). Please verify and correct.

As noted by the reviewer, the jump in references has been verified and corrected

• Dichotomization of Categorical Variables: Provide references that discuss the dichotomization of multiple categorical variables in detail, including the specific statistical methods used instead of the used reference.

As noted by the reviewer, we provided references related to dichotomization of categorical variables in detail in Methods and in discussion section.

• Multivariable vs. Multivariate (Line 132): Use "multivariable" instead of "multivariate" consistently throughout the manuscript, as your model involves one dependent variable and multiple independent variables.

As suggested by the reviewer, we consistently used "multivariable" throughout the manuscript

• Sex vs. Gender: Maintain consistent use of "sex" instead of "gender," given that only clinically relevant variables were collected.

As suggested by the reviewer, we consistently used "sex" instead of "gender," throughout the manuscript

• Missing Participant (Vaccine Type and Age groups): Explain why one participant is missing in the vaccine type variable, and how this relates to the age group stratification.

As noted by the reviewer, we revised the table of participants and vaccine characteristics to rectify any inconsistency. Under Vaccine Type, one participant seemed to be missing because of typing error.

• Bivariate Analysis: Explain why bivariate analysis was not conducted before the multivariable analysis.

Bivariate analysis was conducted but we only presented results from the multivariable logistic regression analysis as we aimed to control for confounding. We controlled for confounding through the use of multivariable analysis. This is described in the Methods, Data Analysis sub-section.

• AEFI Incidence (Lines 180-183): Move the content from lines 180-183 to the incidence of AEFI sub-section of the findings.

As noted by the reviewer, we have moved this content to the incidence of AEFI sub-section of the results.

• Effect Size and Logistic Regression Assumptions: Given the smaller sample size for the Pfizer group compared to the Johnson & Johnson vaccine group, please supplement the analysis with effect size calculations. Alternatively, justify the use of logistic regression by confirming that the data met the necessary assumptions for accurate results.

Though there is a smaller sample size for Pfizer group compared to the Johnson & Johnson vaccine group, there is a minimum number of necessary samples to meet the desired statistical constraints. We controlled f

Attachment Submitted filename: Response to reviewers.doc

# Peer review: Decision Letter 1

**DOI:** 10.1371/journal.pone.0309628.r003

**Authors:** Nuru Abdu, Nuru Abdu

13 Oct 2025

PONE-D-24-31631R1

Adverse events following immunization during COVID-19 mass vaccination campaigns in the Democratic Republic of Congo: Findings from active safety surveillance

PLOS ONE

Dear Dr. Kayumba,

Thank you for submitting your manuscript to PLOS ONE. After careful consideration, we feel that it has merit but does not fully meet PLOS ONE’s publication criteria as it currently stands. Therefore, we invite you to submit a revised version of the manuscript that addresses the points raised during the review process.

Please submit your revised manuscript by Nov 27 2025 11:59PM. If you will need more time than this to complete your revisions, please reply to this message or contact the journal office at plosone@plos.org. When you're ready to submit your revision, log on to https://www.editorialmanager.com/pone/ and select the 'Submissions Needing Revision' folder to locate your manuscript file.

Please include the following items when submitting your revised manuscript:

- A rebuttal letter that responds to each point raised by the academic editor and reviewer(s). You should upload this letter as a separate file labeled 'Response to Reviewers'.
- A marked-up copy of your manuscript that highlights changes made to the original version. You should upload this as a separate file labeled 'Revised Manuscript with Track Changes'.
- An unmarked version of your revised paper without tracked changes. You should upload this as a separate file labeled 'Manuscript'.

-->If you would like to make changes to your financial disclosure, please include your updated statement in your cover letter. Guidelines for resubmitting your figure files are available below the reviewer comments at the end of this letter.

If applicable, we recommend that you deposit your laboratory protocols in protocols.io to enhance the reproducibility of your results. Protocols.io assigns your protocol its own identifier (DOI) so that it can be cited independently in the future. For instructions see: https://journals.plos.org/plosone/s/submission-guidelines#loc-laboratory-protocols. Additionally, PLOS ONE offers an option for publishing peer-reviewed Lab Protocol articles, which describe protocols hosted on protocols.io. Read more information on sharing protocols at https://plos.org/protocols?utm_medium=editorial-email&utm_source=authorletters&utm_campaign=protocols.

We look forward to receiving your revised manuscript.

Kind regards,

Nuru Abdu, BPharm

Academic Editor

PLOS ONE

Journal Requirements:

If the reviewer comments include a recommendation to cite specific previously published works, please review and evaluate these publications to determine whether they are relevant and should be cited. There is no requirement to cite these works unless the editor has indicated otherwise.

Please review your reference list to ensure that it is complete and correct. If you have cited papers that have been retracted, please include the rationale for doing so in the manuscript text, or remove these references and replace them with relevant current references. Any changes to the reference list should be mentioned in the rebuttal letter that accompanies your revised manuscript. If you need to cite a retracted article, indicate the article’s retracted status in the References list and also include a citation and full reference for the retraction notice.

[Note: HTML markup is below. Please do not edit.]

Reviewers' comments:

Reviewer's Responses to Questions

**Comments to the Author**

1. If the authors have adequately addressed your comments raised in a previous round of review and you feel that this manuscript is now acceptable for publication, you may indicate that here to bypass the “Comments to the Author” section, enter your conflict of interest statement in the “Confidential to Editor” section, and submit your "Accept" recommendation.

Reviewer #1: All comments have been addressed

Reviewer #2: (No Response)

Reviewer #3: All comments have been addressed

Reviewer #4: All comments have been addressed

Reviewer #5: (No Response)

**********

2. Is the manuscript technically sound, and do the data support the conclusions?

The manuscript must describe a technically sound piece of scientific research with data that supports the conclusions. Experiments must have been conducted rigorously, with appropriate controls, replication, and sample sizes. The conclusions must be drawn appropriately based on the data presented.

Reviewer #1: Yes

Reviewer #2: Yes

Reviewer #3: Yes

Reviewer #4: Partly

Reviewer #5: Yes

**********

3. Has the statistical analysis been performed appropriately and rigorously?

Reviewer #1: Yes

Reviewer #2: Yes

Reviewer #3: Yes

Reviewer #4: N/A

Reviewer #5: Yes

**********

4. Have the authors made all data underlying the findings in their manuscript fully available?

The PLOS Data policy requires authors to make all data underlying the findings described in their manuscript fully available without restriction, with rare exception (please refer to the Data Availability Statement in the manuscript PDF file). The data should be provided as part of the manuscript or its supporting information, or deposited to a public repository. For example, in addition to summary statistics, the data points behind means, medians and variance measures should be available. If there are restrictions on publicly sharing data—e.g. participant privacy or use of data from a third party—those must be specified.

Reviewer #1: Yes

Reviewer #2: Yes

Reviewer #3: Yes

Reviewer #4: (No Response)

Reviewer #5: Yes

**********

5. Is the manuscript presented in an intelligible fashion and written in standard English?

PLOS ONE does not copyedit accepted manuscripts, so the language in submitted articles must be clear, correct, and unambiguous. Any typographical or grammatical errors should be corrected at revision, so please note any specific errors here.

Reviewer #1: Yes

Reviewer #2: Yes

Reviewer #3: Yes

Reviewer #4: (No Response)

Reviewer #5: Yes

**********

6. Review Comments to the Author

Please use the space provided to explain your answers to the questions above. You may also include additional comments for the author, including concerns about dual publication, research ethics, or publication ethics. (Please upload your review as an attachment if it exceeds 20,000 characters)

Reviewer #1: After detailed processing of the manuscript, taking into account the comments of the reviewers, an interesting study turned out with a logical construction of phrases and reasonable data obtained. All the questions were answered and reflected in the text of the article. One comment appeared regarding the paragraph "Findings from this study showed that the most common adverse event experienced among the majority after COVID-19 vaccination included fever (286 line) (458 cases), pain on injection site (429 cases), (287 line) headache (196 cases), stiffness (72 cases), and myalgia (55 cases). It is advisable for the authors to indicate the % of cases, since % is a more informative indicator for analysis.

Reviewer #2: I reviewed the revised manuscript and I have no additional comments, all the comments have been addressed satisfactorily.

Reviewer #3: Very interesting article, nice to see this studies conducted since they are essential to ensure vaccine safety. All comments from previous reviewers as well as the editor's were addressed. My only comment would be that one of the reviewers suggested as a possible cause for the lower % of AEFIs reported compared to other studies may be the likelihood of previous exposure. While this is certainly a possible explanation, the authors have included it now as the most likely explanation in the discussion while it seems based on the Ghana study that premedication with paracetamol may be just as likely.

Reviewer #4: This may be of worth posting on the governmental institution's website or on their publications. However, I do not believe it is worthy of publication as an article in a peer-reviewed journal that is read by people all over the world.

This is because, although the risks of short-term, relatively common adverse reactions to the SARS-CoV-2 vaccine varies depending on the individual circumstances of the subjects, they do not much vary between countries around the world.

Therefore, if the risks of adverse reactions in the authors’ country are significantly and greatly different from that of generally seen in other countries with careful discussion on the reasons of difference, it may be worthy of publication as an article in a peer-reviewed journal.

However, unless they cound such difference with thoughtful discussion, I do not believe it is worthy of publication as an article in a peer-reviewed journal.

I could neither find such difference, consequently nore careful discussions in this manuscript.

Reviewer #5: General impression

The manuscript addresses a highly relevant topic for public health, particularly considering the underrepresentation of African populations in previous COVID-19 vaccine trials. The active safety surveillance methodology strengthens the study and provides valuable evidence on adverse events following immunization in the Democratic Republic of Congo.

Strengths

Large and well-described sample (n=4766).

Active follow-up up to 28 days, improving data quality.

Inclusion of analyses by age group and vaccine type.

Discussion well contextualized with international literature.

Major comments

Clarity of English writing – Despite improvements, some sections (particularly Introduction and Discussion) remain difficult to read. A professional language revision is recommended.

Methodology – Participant selection – Clarify potential biases (e.g., exclusion of individuals without phone access), as this may affect representativeness.

Discussion – Pre-medication – The absence of paracetamol use assessment should be highlighted as a formal limitation.

Rare adverse events – Specify whether unusual AEFIs (e.g., menstrual disturbances) were clinically verified or self-reported, as this impacts robustness of conclusions.

Supplementary data – Provide full tables including less frequent AEFIs as supplementary material for transparency.

Minor comments

Adjust minor formatting inconsistencies in tables.

Standardize terminology (use “AEFI” consistently).

Reduce redundancy between Results and Discussion.

Conclusion

This is a valuable study that would benefit from:

additional clarity in the text,

more detail on potential selection bias,

clear acknowledgment of limitations,

clarification of validation of rare AEFIs.

I recommend minor to moderate revision before publication.

**********

7. PLOS authors have the option to publish the peer review history of their article (what does this mean?). If published, this will include your full peer review and any attached files.

If you choose “no”, your identity will remain anonymous but your review may still be made public.

**Do you want your identity to be public for this peer review?**  For information about this choice, including consent withdrawal, please see our Privacy Policy.

Reviewer #1: **Yes:** Mikhail P. Kostinov

Reviewer #2: No

Reviewer #3: **Yes:** Margarita Riera-Montes

Reviewer #4: No

Reviewer #5: **Yes:** Clarissa Leite Santos

**********

[NOTE: If reviewer comments were submitted as an attachment file, they will be attached to this email and accessible via the submission site. Please log into your account, locate the manuscript record, and check for the action link "View Attachments". If this link does not appear, there are no attachment files.]

While revising your submission, please upload your figure files to the Preflight Analysis and Conversion Engine (PACE) digital diagnostic tool, https://pacev2.apexcovantage.com/. PACE helps ensure that figures meet PLOS requirements. To use PACE, you must first register as a user. Registration is free. Then, login and navigate to the UPLOAD tab, where you will find detailed instructions on how to use the tool. If you encounter any issues or have any questions when using PACE, please email PLOS at figures@plos.org. Please note that Supporting Information files do not need this step.

Attachment Submitted filename: Review result.docx

# Author comment: Author response to Decision Letter 2

**DOI:** 10.1371/journal.pone.0309628.r004

27 Oct 2025

October 27, 2025

Dear Nuru Abdu

Enclosed is a revised version of our manuscript, titled: “Adverse events following immunization during COVID-19 mass vaccination campaigns in the Democratic Republic of Congo: Findings from active safety surveillance,” for your renewed consideration as an original contribution in the Journal. As suggested by reviewers, we have addressed the comments from reviewers and included their insights in our manuscript.

Thank you for the opportunity to improve our manuscript by responding to the comments of the reviewers. Below we respond to specific comments and queries.

We look forward to your response.

Yours sincerely,

Kizito Kayumba, M.D., MSc

Africa Centers for Disease Control and Prevention

Addis Ababa, Ethiopia

Ph: +250 788 30 1834

KizitoK@africacdc.org

Journal Requirements:

If the reviewer comments include a recommendation to cite specific previously published works, please review and evaluate these publications to determine whether they are relevant and should be cited. There is no requirement to cite these works unless the editor has indicated otherwise.

Thank you for this insight, the reviewer comments do not include any recommendation to cite a specific previously published works

Please review your reference list to ensure that it is complete and correct. If you have cited papers that have been retracted, please include the rationale for doing so in the manuscript text, or remove these references and replace them with relevant current references. Any changes to the reference list should be mentioned in the rebuttal letter that accompanies your revised manuscript. If you need to cite a retracted article, indicate the article’s retracted status in the References list and also include a citation and full reference for the retraction notice.

We thank the editor for drawing our attention to this. We have reviewed our reference list. We do not have any references that have been retracted. However, we made some changes to the reference list to address some typos errors and journal abbreviations:

On reference 2: We rectified a typos error in the title of the reference to become “ Vaccine-induced immune thrombotic thrombocytopenia (VITT) with massive pulmonary embolism and thrombocytopenia following a single dose of Janssen Ad26.COV2.S vaccination. Int J Infect Dis. 2022;116:154-156.” Instead of “ A Vaccine-induced massive pulmonary embolism and thrombocytopenia following a single dose of Janssen Ad26.COV2.S vaccination.”

On Reference 6: We replaced the abbreviation “WHO” by “World Health Organization” in the reference to be consistent with the journal abbreviations standard

[Note: HTML markup is below. Please do not edit.]

Reviewers' comments:

Reviewer's Responses to Questions

Comments to the Author

1. If the authors have adequately addressed your comments raised in a previous round of review and you feel that this manuscript is now acceptable for publication, you may indicate that here to bypass the “Comments to the Author” section, enter your conflict of interest statement in the “Confidential to Editor” section, and submit your "Accept" recommendation.

Reviewer #1: All comments have been addressed

Reviewer #2: (No Response)

Reviewer #3: All comments have been addressed

Reviewer #4: All comments have been addressed

Reviewer #5: (No Response)

________________________________________

2. Is the manuscript technically sound, and do the data support the conclusions?

The manuscript must describe a technically sound piece of scientific research with data that supports the conclusions. Experiments must have been conducted rigorously, with appropriate controls, replication, and sample sizes. The conclusions must be drawn appropriately based on the data presented.

Reviewer #1: Yes

Reviewer #2: Yes

Reviewer #3: Yes

Reviewer #4: Partly

Reviewer #5: Yes

________________________________________

3. Has the statistical analysis been performed appropriately and rigorously?

Reviewer #1: Yes

Reviewer #2: Yes

Reviewer #3: Yes

Reviewer #4: N/A

Reviewer #5: Yes

________________________________________

4. Have the authors made all data underlying the findings in their manuscript fully available?

The PLOS Data policy requires authors to make all data underlying the findings described in their manuscript fully available without restriction, with rare exception (please refer to the Data Availability Statement in the manuscript PDF file). The data should be provided as part of the manuscript or its supporting information, or deposited to a public repository. For example, in addition to summary statistics, the data points behind means, medians and variance measures should be available. If there are restrictions on publicly sharing data—e.g. participant privacy or use of data from a third party—those must be specified.

Reviewer #1: Yes

Reviewer #2: Yes

Reviewer #3: Yes

Reviewer #4: (No Response)

Reviewer #5: Yes

________________________________________

5. Is the manuscript presented in an intelligible fashion and written in standard English?

PLOS ONE does not copyedit accepted manuscripts, so the language in submitted articles must be clear, correct, and unambiguous. Any typographical or grammatical errors should be corrected at revision, so please note any specific errors here.

Reviewer #1: Yes

Reviewer #2: Yes

Reviewer #3: Yes

Reviewer #4: (No Response)

Reviewer #5: Yes

________________________________________

6. Review Comments to the Author

Please use the space provided to explain your answers to the questions above. You may also include additional comments for the author, including concerns about dual publication, research ethics, or publication ethics. (Please upload your review as an attachment if it exceeds 20,000 characters)

Reviewer #1: After detailed processing of the manuscript, taking into account the comments of the reviewers, an interesting study turned out with a logical construction of phrases and reasonable data obtained. All the questions were answered and reflected in the text of the article. One comment appeared regarding the paragraph "Findings from this study showed that the most common adverse event experienced among the majority after COVID-19 vaccination included fever (286 line) (458 cases), pain on injection site (429 cases), (287 line) headache (196 cases), stiffness (72 cases), and myalgia (55 cases). It is advisable for the authors to indicate the % of cases, since % is a more informative indicator for analysis.

We thank the reviewer for this valuable comment. We have expressed the incidence of these AEFIs in percentage

Reviewer #2: I reviewed the revised manuscript and I have no additional comments, all the comments have been addressed satisfactorily.

Reviewer #3: Very interesting article, nice to see this studies conducted since they are essential to ensure vaccine safety. All comments from previous reviewers as well as the editor's were addressed. My only comment would be that one of the reviewers suggested as a possible cause for the lower % of AEFIs reported compared to other studies may be the likelihood of previous exposure. While this is certainly a possible explanation, the authors have included it now as the most likely explanation in the discussion while it seems based on the Ghana study that premedication with paracetamol may be just as likely.

We thank the reviewer for this comment. As we have not assessed for premedication with paracetamol, we hypothesized that this may mask AEFI based on the Ghana study. However, previous exposure could have been impacted ton AEFI incidence as well, as the study participants had been exposed to the virus before vaccination. We kept the two hypotheses in our discussion and suggested for more evidence to explain these findings.

Reviewer #4: This may be of worth posting on the governmental institution's website or on their publications. However, I do not believe it is worthy of publication as an article in a peer-reviewed journal that is read by people all over the world.

This is because, although the risks of short-term, relatively common adverse reactions to the SARS-CoV-2 vaccine varies depending on the individual circumstances of the subjects, they do not much vary between countries around the world.

Therefore, if the risks of adverse reactions in the authors’ country are significantly and greatly different from that of generally seen in other countries with careful discussion on the reasons of difference, it may be worthy of publication as an article in a peer-reviewed journal.

However, unless they cound such difference with thoughtful discussion, I do not believe it is worthy of publication as an article in a peer-reviewed journal.

I could neither find such difference, consequently nore careful discussions in this manuscript.

We thank the reviewer for their thoughtful critique. We respectfully disagree with the assessment and believe that our manuscript makes a critical contribution to the global literature, precisely by addressing the significant evidence gap in pharmacovigilance for underrepresented populations.

The reviewer suggests that AEFI risks do not vary greatly between countries. However, we are convinced that while the types of AEFIs may be similar, their incidence, severity, and risk factors cannot be assumed identical across different genetic and environmental backgrounds. Our study identifies a substantial and important difference: the overall AEFI incidence in our DRC cohort was 23.75%, which is markedly lower than the 65-80% reported in many Western studies (e.g., UK and US participant-reported data). This finding is not merely a minor variation but a significant discrepancy that warrants international attention and explanation.

In our discussion, we provide a careful and thoughtful analysis of potential reasons for this difference, directly addressing the reviewer's request. We hypothesize that a high rate of prior, undocumented SARS-CoV-2 infection in the DRC population may have led to a better-tolerated, non-primary immune response. We also cite regional practices, such as the widespread pre-medication with analgesics documented in the region, and methodological differences between our active surveillance and passive, app-based reporting systems that may over-represent AEFIs. This lower incidence has direct implications for public health communication and vaccine confidence in the region.

Beyond this key finding, the manuscript's value is multi-faceted. It provides the first robust, active safety surveillance data for a mass vaccination campaign in the DRC, filling a critical gap for a population underrepresented in initial clinical trials. It also offers locally relevant evidence on risk factors, demonstrating significantly lower odds of AEFIs in both the very young and elderly within this specific demographic. The strongly decreased odds of AEFIs in those under 18 and over 60 provides reassuring local safety data for vulnerable age groups, supporting continued vaccination efforts for the elderly and paving the way for pediatric vaccination campaigns. Finally, it serves as a proof-of-concept for implementing advanced pharmacovigilance in a low-resource setting, a valuable methodological contribution for global health security.

We are confident that with revisions that more prominently highlight these comparative findings and their implications, the manuscript will be seen as a worthy contribution to the peer-reviewed literature, providing essential safety data for a global audience.

Reviewer #5: General impression

The manuscript addresses a highly relevant topic for public health, particularly considering the underrepresentation of African populations in previous COVID-19 vaccine trials. The active safety surveillance methodology strengthens the study and provides valuable evidence on adverse events following immunization in the Democratic Republic of Congo.

Strengths

Large and well-described sample (n=4766).

Active follow-up up to 28 days, improving data quality.

Inclusion of analyses by age group and vaccine type.

Discussion well contextualized with international literature.

Major comments

Clarity of English writing – Despite improvements, some sections (particularly Introduction and Discussion) remain difficult to read. A professional language revision is recommended.

We thank the reviewer for this thorough observation and recommendation. We have reviewed the introduction and discussion with a professional English language to make these sections easy to read.

Methodology – Participant selection – Clarify potential biases (e.g., exclusion of individuals without phone access), as this may affect representativeness.

We thank the reviewer and we concur with their observation that excluding individuals without phone access could constitute a selection bias, affecting representativeness. We have highlighted this in the manuscript, in the section of Limitations

Discussion – Pre-medication – The absence of paracetamol use assessment should be highlighted as a formal limitation.

We appreciate the suggestion of the reviewer to mention the absence of paracetamol use assessment as a formal limitation. We have highlighted this in the section of study limitations.

Rare adverse events – Specify whether unusual AEFIs (e.g., menstrual disturbances) were clinically verified or self-reported, as this impacts robustness of conclusions.

We thank the reviewer for emphasizing on how unusual adverse events were assessed. These were not clinically verified, which impacts robustness of conclusions. We have specified this in the limitation section as well.

Supplementary data – Provide full tables including less frequent AEFIs as supplementary material for transparency.

We appreciate the suggestion from reviewer to provide tables including less frequent AEFIs. We have included the tables of less frequent AEFIs in S1 Table of Supplementary material.

Minor comments

Adjust minor formatting inconsistencies in tables.

We thank the reviewer for this observation. We have thoroughly reviewed the tables and addressed formatting inconsistencies

Standardize terminology (use “AEFI” consistently).

We appreciate this comment and we have standardized the use of AEFI consistently

Reduce redundancy between Results and Discussion.

We thank the reviewer for this great insight. We managed to reduce redundancy between Results and Discussion by removing from discussion the information that was already provided in Results Section. We reshaped the Discussion Section to improve clarity and enable easy reading

Attachment Submitted filename: Responses to Reviewers comments.pdf

# Peer review: Decision Letter 2

**DOI:** 10.1371/journal.pone.0309628.r005

**Authors:** Nuru Abdu, Nuru Abdu, Nuru Abdu

30 Dec 2025

PONE-D-24-31631R2

Adverse events following immunization during COVID-19 mass vaccination campaigns in the Democratic Republic of Congo: Findings from active safety surveillance

PLOS One

Dear Dr. Kayumba,

Thank you for submitting your manuscript to PLOS ONE. After careful consideration, we feel that it has merit but does not fully meet PLOS ONE’s publication criteria as it currently stands. Therefore, we invite you to submit a revised version of the manuscript that addresses the points raised during the review process.

Please submit your revised manuscript by Feb 13 2026 11:59PM. If you will need more time than this to complete your revisions, please reply to this message or contact the journal office at plosone@plos.org. When you're ready to submit your revision, log on to https://www.editorialmanager.com/pone/ and select the 'Submissions Needing Revision' folder to locate your manuscript file.

Please include the following items when submitting your revised manuscript:

- A letter that responds to each point raised by the academic editor and reviewer(s). You should upload this letter as a separate file labeled 'Response to Reviewers'.
- A marked-up copy of your manuscript that highlights changes made to the original version. You should upload this as a separate file labeled 'Revised Manuscript with Track Changes'.
- An unmarked version of your revised paper without tracked changes. You should upload this as a separate file labeled 'Manuscript'.

If you would like to make changes to your financial disclosure, please include your updated statement in your cover letter. Guidelines for resubmitting your figure files are available below the reviewer comments at the end of this letter.

If applicable, we recommend that you deposit your laboratory protocols in protocols.io to enhance the reproducibility of your results. Protocols.io assigns your protocol its own identifier (DOI) so that it can be cited independently in the future. For instructions see: https://journals.plos.org/plosone/s/submission-guidelines#loc-laboratory-protocols. Additionally, PLOS ONE offers an option for publishing peer-reviewed Lab Protocol articles, which describe protocols hosted on protocols.io. Read more information on sharing protocols at https://plos.org/protocols?utm_medium=editorial-email&utm_source=authorletters&utm_campaign=protocols.

We look forward to receiving your revised manuscript.

Kind regards,

Nuru Abdu, BPharm

Academic Editor

PLOS One

Journal Requirements:

If the reviewer comments include a recommendation to cite specific previously published works, please review and evaluate these publications to determine whether they are relevant and should be cited. There is no requirement to cite these works unless the editor has indicated otherwise.

[Note: HTML markup is below. Please do not edit.]

Reviewers' comments:

Reviewer's Responses to Questions

**Comments to the Author**

1. If the authors have adequately addressed your comments raised in a previous round of review and you feel that this manuscript is now acceptable for publication, you may indicate that here to bypass the “Comments to the Author” section, enter your conflict of interest statement in the “Confidential to Editor” section, and submit your "Accept" recommendation.

Reviewer #1: All comments have been addressed

Reviewer #2: (No Response)

Reviewer #6: (No Response)

**********

2. Is the manuscript technically sound, and do the data support the conclusions?

The manuscript must describe a technically sound piece of scientific research with data that supports the conclusions. Experiments must have been conducted rigorously, with appropriate controls, replication, and sample sizes. The conclusions must be drawn appropriately based on the data presented.

Reviewer #1: Yes

Reviewer #2: Yes

Reviewer #6: Yes

**********

3. Has the statistical analysis been performed appropriately and rigorously?

Reviewer #1: Yes

Reviewer #2: Yes

Reviewer #6: Yes

**********

4. Have the authors made all data underlying the findings in their manuscript fully available?

The PLOS Data policy requires authors to make all data underlying the findings described in their manuscript fully available without restriction, with rare exception (please refer to the Data Availability Statement in the manuscript PDF file). The data should be provided as part of the manuscript or its supporting information, or deposited to a public repository. For example, in addition to summary statistics, the data points behind means, medians and variance measures should be available. If there are restrictions on publicly sharing data—e.g. participant privacy or use of data from a third party—those must be specified.

Reviewer #1: Yes

Reviewer #2: Yes

Reviewer #6: Yes

**********

5. Is the manuscript presented in an intelligible fashion and written in standard English?

PLOS ONE does not copyedit accepted manuscripts, so the language in submitted articles must be clear, correct, and unambiguous. Any typographical or grammatical errors should be corrected at revision, so please note any specific errors here.

Reviewer #1: Yes

Reviewer #2: Yes

Reviewer #6: Yes

**********

6. Review Comments to the Author

Please use the space provided to explain your answers to the questions above. You may also include additional comments for the author, including concerns about dual publication, research ethics, or publication ethics. (Please upload your review as an attachment if it exceeds 20,000 characters)

Reviewer #1: All the existing comments were eliminated during the previous iterations of the review. There are no questions about the current edition of the manuscript

Reviewer #2: I no additional comments for the authors. However, the comments raised by reviewer no. 4 are critical.

Reviewer #6: It is an interesting article; the methodology in which ad-hoc questionnaires on safety are conducted complements the data obtained through spontaneous reporting, favoring the acquisition of the complete safety profile of the drug when used under real conditions. The safety of vaccines used in the prevention of COVID is another relevant aspect. This methodology also allows us to understand how different variables affect the safety profile of these vaccines, such as age, the number of doses, and the time since administration.

Methodology.

The low representativeness of patients under 18 years and over 65 years may be due to the type of recruitment through a telephone intervention, and this should be noted in the study's limitations.

Include the questionnaire in supplementary materials, is there any section for the patient's free response?, what methodology was followed for its development?

Results

The sample is small in patients with the BNT162b2 vaccine and in age groups over 65 years and under 18 years; it would also be interesting to know the incidence of a second vaccine dose or heterologous vaccination. Has repeating the intervention at a later time to obtain this data been considered?

The age range between 18 and 65 years is too wide; a more detailed analysis would allow seeing the reactivity in young patients compared to middle-aged patients.

Discussion

Comment on the spontaneous reporting data in this country regarding these medications.

Include other studies with a similar methodology that allows knowing the safety profile of other medications.

Discuss the advantages and disadvantages of a telephone intervention compared to an in-person one, and what possibilities there would be to carry it out in this context, for example, in community pharmacies.

**********

7. PLOS authors have the option to publish the peer review history of their article (what does this mean?). If published, this will include your full peer review and any attached files.

If you choose “no”, your identity will remain anonymous but your review may still be made public.

**Do you want your identity to be public for this peer review?**  For information about this choice, including consent withdrawal, please see our Privacy Policy.

Reviewer #1: **Yes:** Mikhail P. Kostinov

Reviewer #2: No

Reviewer #6: No

**********

[NOTE: If reviewer comments were submitted as an attachment file, they will be attached to this email and accessible via the submission site. Please log into your account, locate the manuscript record, and check for the action link "View Attachments". If this link does not appear, there are no attachment files.]

To ensure your figures meet our technical requirements, please review our figure guidelines: https://journals.plos.org/plosone/s/figures

You may also use PLOS’s free figure tool, NAAS, to help you prepare publication quality figures: https://journals.plos.org/plosone/s/figures#loc-tools-for-figure-preparation.

NAAS will assess whether your figures meet our technical requirements by comparing each figure against our figure specifications.

# Author comment: Author response to Decision Letter 3

**DOI:** 10.1371/journal.pone.0309628.r006

12 Feb 2026

Journal Requirements:

If the reviewer comments include a recommendation to cite specific previously published works, please review and evaluate these publications to determine whether they are relevant and should be cited. There is no requirement to cite these works unless the editor has indicated otherwise.

Thank you for this insight. The reviewer comments do not include any recommendation to cite a specific previously published works

Comments to the Author

1. If the authors have adequately addressed your comments raised in a previous round of review and you feel that this manuscript is now acceptable for publication, you may indicate that here to bypass the “Comments to the Author” section, enter your conflict of interest statement in the “Confidential to Editor” section, and submit your "Accept" recommendation.

Reviewer #1: All comments have been addressed

Reviewer #2: (No Response)

Reviewer #6: (No Response)

________________________________________

2. Is the manuscript technically sound, and do the data support the conclusions?

The manuscript must describe a technically sound piece of scientific research with data that supports the conclusions. Experiments must have been conducted rigorously, with appropriate controls, replication, and sample sizes. The conclusions must be drawn appropriately based on the data presented.

Reviewer #1: Yes

Reviewer #2: Yes

Reviewer #6: Yes

________________________________________

3. Has the statistical analysis been performed appropriately and rigorously?

Reviewer #1: Yes

Reviewer #2: Yes

Reviewer #6: Yes

________________________________________

4. Have the authors made all data underlying the findings in their manuscript fully available?

The PLOS Data policy requires authors to make all data underlying the findings described in their manuscript fully available without restriction, with rare exception (please refer to the Data Availability Statement in the manuscript PDF file). The data should be provided as part of the manuscript or its supporting information, or deposited to a public repository. For example, in addition to summary statistics, the data points behind means, medians and variance measures should be available. If there are restrictions on publicly sharing data—e.g. participant privacy or use of data from a third party—those must be specified.

Reviewer #1: Yes

Reviewer #2: Yes

Reviewer #6: Yes

________________________________________

5. Is the manuscript presented in an intelligible fashion and written in standard English?

PLOS ONE does not copyedit accepted manuscripts, so the language in submitted articles must be clear, correct, and unambiguous. Any typographical or grammatical errors should be corrected at revision, so please note any specific errors here.

Reviewer #1: Yes

Reviewer #2: Yes

Reviewer #6: Yes

________________________________________

6. Review Comments to the Author

Reviewer #1: All the existing comments were eliminated during the previous iterations of the review. There are no questions about the current edition of the manuscript

We thank the reviewer for confirming that all previous comments have been satisfactorily addressed.

Reviewer #2: I no additional comments for the authors. However, the comments raised by reviewer no. 4 are critical.

We thank the reviewer for this assessment. The comments raised by the Reviewer have been addressed in the revised manuscript, with clarifications and additions incorporated into the Discussion section.

Reviewer #6: It is an interesting article; the methodology in which ad-hoc questionnaires on safety are conducted complements the data obtained through spontaneous reporting, favoring the acquisition of the complete safety profile of the drug when used under real conditions. The safety of vaccines used in the prevention of COVID is another relevant aspect. This methodology also allows us to understand how different variables affect the safety profile of these vaccines, such as age, the number of doses, and the time since administration.

We thank the reviewer for this positive and insightful evaluation of our work. We agree that active safety surveillance using structured questionnaires complements spontaneous reporting systems and provides a more comprehensive real-world safety profile.

Methodology.

1) The low representativeness of patients under 18 years and over 65 years may be due to the type of recruitment through a telephone intervention, and this should be noted in the study's limitations.

We agree with the reviewer. As suggested, we have explicitly acknowledged this limitation in the Limitations section, noting that the telephone-based recruitment and follow-up approach may have introduced selection bias and reduced representativeness among younger and older age groups (<18 years and ≥60 years).

2) Include the questionnaire in supplementary materials, is there any section for the patient's free response? what methodology was followed for its development?

We thank the reviewer for this important suggestion. The questionnaire has been included as Supporting Information (S4 Table).The questionnaire was WHO-adapted with pre-defined AESIs and included an open field for additional participant-reported AEFIs (others to be specified), which were coded prior to analysis. We clarified this in the Data collection and management subsection

Results

3) The sample is small in patients with the BNT162b2 vaccine and in age groups over 65 years and under 18 years; it would also be interesting to know the incidence of a second vaccine dose or heterologous vaccination. Has repeating the intervention at a later time to obtain this data been considered?

We thank the reviewer for this insight. We acknowledge this limitation and we clarified in the Limitations section that the low number of second-dose recipients and absence of heterologous vaccination limited our ability to conduct meaningful subgroup analyses. We have also added a statement noting that future or repeated active surveillance rounds will be required to assess second-dose and heterologous vaccination safety.

4) The age range between 18 and 65 years is too wide; a more detailed analysis would allow seeing the reactivity in young patients compared to middle-aged patients.

We appreciate this suggestion. This point has been acknowledged in the Limitations section, noting that the broad age grouping (18–59 years) may have masked differences between younger and middle-aged adults and should be explored in future studies with larger samples.

Discussion

5) Comment on the spontaneous reporting data in this country regarding these medications.

Include other studies with a similar methodology that allows knowing the safety profile of other medications.

We agree with the reviewer that active safety surveillance methodologies have been widely used to assess vaccine and medication safety beyond COVID-19 vaccines, particularly in post-marketing contexts. We expanded the Discussion and we have added a subsection entitled “Active surveillance as a complement to spontaneous reporting” into the Discussion section where we referenced additional studies using active or cohort-based surveillance approaches (including vaccine safety studies in Africa and other LMICs), highlighting methodological similarities and reinforcing the validity of our approach for evaluating real-world safety profiles.

6) Discuss the advantages and disadvantages of a telephone intervention compared to an in-person one, and what possibilities there would be to carry it out in this context, for example, in community pharmacies.

We appreciate this insightful comment. In the Discussion section, we have included a concise comparison of telephone-based versus in-person surveillance, outlining key advantages (feasibility, cost, scale) and limitations (selection bias, self-reporting, lack of clinical verification), and briefly discussed potential alternative community-based approaches within the DRC context.

We thank all reviewers for their constructive feedback and careful evaluation of the manuscript. We believe that the revisions made have substantially improved the clarity, methodological transparency, and relevance of the study.

Attachment Submitted filename: Responses_to_Reviewers_Comments_auresp_3.pdf

# Peer review: Decision Letter 3

**DOI:** 10.1371/journal.pone.0309628.r007

**Authors:** Nuru Abdu, Nuru Abdu, Nuru Abdu, Mohamed Lounis

18 Jun 2026

Adverse events following immunization during COVID-19 mass vaccination campaigns in the Democratic Republic of Congo: Findings from active safety surveillance

PONE-D-24-31631R3

Dear Dr. Kayumba,

We’re pleased to inform you that your manuscript has been judged scientifically suitable for publication and will be formally accepted for publication once it meets all outstanding technical requirements.

Within one week, you’ll receive an e-mail detailing the required amendments. When these have been addressed, you’ll receive a formal acceptance letter and your manuscript will be scheduled for publication.

An invoice will be generated when your article is formally accepted. Please note, if your institution has a publishing partnership with PLOS and your article meets the relevant criteria, all or part of your publication costs will be covered. Please make sure your user information is up-to-date by logging into Editorial Manager at Editorial Manager® and clicking the ‘Update My Information' link at the top of the page. For questions related to billing, please contact billing support.

If your institution or institutions have a press office, please notify them about your upcoming paper to help maximize its impact. If they’ll be preparing press materials, please inform our press team as soon as possible -- no later than 48 hours after receiving the formal acceptance. Your manuscript will remain under strict press embargo until 2 pm Eastern Time on the date of publication. For more information, please contact onepress@plos.org.

Kind regards,

Mohamed Lounis

Academic Editor

PLOS One

Additional Editor Comments (optional):

The authors have adressed all reviewers comments.

Reviewers' comments:

Reviewer's Responses to Questions

**Comments to the Author**

1. If the authors have adequately addressed your comments raised in a previous round of review and you feel that this manuscript is now acceptable for publication, you may indicate that here to bypass the “Comments to the Author” section, enter your conflict of interest statement in the “Confidential to Editor” section, and submit your "Accept" recommendation.

Reviewer #1: All comments have been addressed

Reviewer #6: All comments have been addressed

**********

2. Is the manuscript technically sound, and do the data support the conclusions?

The manuscript must describe a technically sound piece of scientific research with data that supports the conclusions. Experiments must have been conducted rigorously, with appropriate controls, replication, and sample sizes. The conclusions must be drawn appropriately based on the data presented.

Reviewer #1: Yes

Reviewer #6: Yes

**********

3. Has the statistical analysis been performed appropriately and rigorously?

Reviewer #1: Yes

Reviewer #6: Yes

**********

4. Have the authors made all data underlying the findings in their manuscript fully available?

The PLOS Data policy requires authors to make all data underlying the findings described in their manuscript fully available without restriction, with rare exception (please refer to the Data Availability Statement in the manuscript PDF file). The data should be provided as part of the manuscript or its supporting information, or deposited to a public repository. For example, in addition to summary statistics, the data points behind means, medians and variance measures should be available. If there are restrictions on publicly sharing data—e.g. participant privacy or use of data from a third party—those must be specified.

Reviewer #1: Yes

Reviewer #6: Yes

**********

5. Is the manuscript presented in an intelligible fashion and written in standard English?

PLOS ONE does not copyedit accepted manuscripts, so the language in submitted articles must be clear, correct, and unambiguous. Any typographical or grammatical errors should be corrected at revision, so please note any specific errors here.

Reviewer #1: Yes

Reviewer #6: Yes

**********

6. Review Comments to the Author

Please use the space provided to explain your answers to the questions above. You may also include additional comments for the author, including concerns about dual publication, research ethics, or publication ethics. (Please upload your review as an attachment if it exceeds 20,000 characters)

Reviewer #1: (No Response)

Reviewer #6: (No Response)

**********

7. PLOS authors have the option to publish the peer review history of their article (what does this mean?). If published, this will include your full peer review and any attached files.

If you choose “no”, your identity will remain anonymous but your review may still be made public.

**Do you want your identity to be public for this peer review?**  For information about this choice, including consent withdrawal, please see our Privacy Policy.

Reviewer #1: **Yes:** Mikhail P. Kostinov

Reviewer #6: No

**********

# Editor report: Acceptance letter

**DOI:** 10.1371/journal.pone.0309628.r008

**Authors:** Nuru Abdu, Nuru Abdu, Nuru Abdu, Mohamed Lounis

PONE-D-24-31631R3

PLOS One

Dear Dr. Kayumba,

I'm pleased to inform you that your manuscript has been deemed suitable for publication in PLOS One. Congratulations! Your manuscript is now being handed over to our production team.

At this stage, our production department will prepare your paper for publication. This includes ensuring the following:

* All references, tables, and figures are properly cited

* All relevant supporting information is included in the manuscript submission,

* There are no issues that prevent the paper from being properly typeset

You will receive further instructions from the production team, including instructions on how to review your proof when it is ready. Please keep in mind that we are working through a large volume of accepted articles, so please give us a few days to review your paper and let you know the next and final steps.

Lastly, if your institution or institutions have a press office, please let them know about your upcoming paper now to help maximize its impact. If they'll be preparing press materials, please inform our press team within the next 48 hours. Your manuscript will remain under strict press embargo until 2 pm Eastern Time on the date of publication. For more information, please contact onepress@plos.org.

You will receive an invoice from PLOS for your publication fee after your manuscript has reached the completed accept phase. If you receive an email requesting payment before acceptance or for any other service, this may be a phishing scheme. Learn how to identify phishing emails and protect your accounts at https://explore.plos.org/phishing.

If we can help with anything else, please email us at customercare@plos.org.

Thank you for submitting your work to PLOS ONE and supporting open access.

Kind regards,

PLOS ONE Editorial Office Staff

on behalf of

Dr. Mohamed Lounis

Academic Editor

PLOS One
